# Expression and activity of the calcitonin receptor family in a sample of primary human high-grade gliomas

**DOI:** 10.1186/s12885-019-5369-y

**Published:** 2019-02-18

**Authors:** Anna Ostrovskaya, Caroline Hick, Dana S. Hutchinson, Brett W. Stringer, Peter J. Wookey, Denise Wootten, Patrick M. Sexton, Sebastian G. B. Furness

**Affiliations:** 1 Drug Discovery Biology and Department of Pharmacology, Monash Institute of Pharmaceutical Sciences, Monash University, Parkville, VIC 3052 Australia; 2 QIMR-Berghofer Medical Research Institute, Brisbane, QLD Australia; 3 Department of Medicine/Cardiology (Austin Health, Heidelberg), University of Melbourne, Lance Townsend Building, Level 10, Austin Campus, Studley Road, Heidelberg, VIC 3084 Australia; 4 School of Pharmacy, Fudan University, Shanghai, 201203 China

**Keywords:** Calcitonin receptor, Glioblastoma, GPCR, Signaling

## Abstract

**Background:**

Glioblastoma (GBM) is the most common and aggressive type of primary brain cancer. With median survival of less than 15 months, identification and validation of new GBM therapeutic targets is of critical importance.

**Results:**

In this study we tested expression and performed pharmacological characterization of the calcitonin receptor (CTR) as well as other members of the calcitonin family of receptors in high-grade glioma (HGG) cell lines derived from individual patient tumours, cultured in defined conditions.

Previous immunohistochemical data demonstrated CTR expression in GBM biopsies and we were able to confirm CALCR (gene encoding CTR) expression. However, as assessed by cAMP accumulation assay, only one of the studied cell lines expressed functional CTR, while the other cell lines have functional CGRP (CLR/RAMP1) receptors. The only CTR-expressing cell line (SB2b) showed modest coupling to the cAMP pathway and no activation of other known CTR signaling pathways, including ERK_1/2_ and p38 MAP kinases, and Ca^2+^ mobilization, supportive of low cell surface receptor expression.

Exome sequencing data failed to account for the discrepancy between functional data and expression on the cell lines that do not respond to calcitonin(s) with no deleterious non-synonymous polymorphisms detected, suggesting that other factors may be at play, such as alternative splicing or rapid constitutive receptor internalisation.

**Conclusions:**

This study shows that GPCR signaling can display significant variation depending on cellular system used, and effects seen in model recombinant cell lines or tumour cell lines are not always reproduced in a more physiologically relevant system and vice versa.

**Electronic supplementary material:**

The online version of this article (10.1186/s12885-019-5369-y) contains supplementary material, which is available to authorized users.

## Background

Glioblastoma (GBM) is the most common and aggressive type of primary brain cancer [1, 2]. GBM arises from transformed precursors of astrocyte-glial lineage [3] and is characterized by high proliferation, vascularization and resistance to apoptosis. GBM have been classified into four subtypes: proneural, neural, classical and mesenchymal based on distinct genomic and proteomic profiles, and these subtypes have distinct responsiveness to the existing combined therapy protocol [4]. GBMs are highly heterogeneous tumours, comprising cells in various states of differentiation including a subpopulation of cells that display stem cell characteristics [3]. Excessive blood vessel formation resulting from hypoxia, secretion of angiogenic growth factors by GBM cells and glioma stem cell differentiation into endothelial cells and pericyte precursors (vascular mimicry) contributes to rapid disease progression [5–9]. With median survival of less than 15 months, even with best practice intervention, identification and validation of new GBM therapeutic targets is of critical importance.

The calcitonin family of receptors consist of the calcitonin receptor (gene:CALCR, protein:CTR) and the calcitonin receptor-like receptor (gene:CALRL, protein:CLR); both are class B (or secretin-like) G protein-coupled receptors (GPCRs). CTR and CLR can form complexes with the accessory single-transmembrane-domain proteins known as the receptor activity-modifying proteins (RAMPs) [10] generating multiple distinct receptor phenotypes with different specificities for the calcitonin (CT) peptide family (Table 1) [11].

**Table 1:** Calcitonin family of receptors [10, 11, 38]

Receptor	Heteromers	Peptides rank order of potency (human)
CTR	CTR	*sCT ≥ hCT > AMY, αCGRP > AM, intermedin*
AMY1	CTR + RAMP1	*sCT ≥ AMY ≥ αCGRP > intermedin ≥ hCT > AM*
AMY2	CTR + RAMP2	*Poorly defined*
AMY3	CTR + RAMP3	*sCT ≥ AMY > αCGRP > intermedin ≥ hCT > AM*
CGRP	CLR + RAMP1	*αCGRP > AM ≥ intermedin > AMY ≥ sCT*
AM1	CLR + RAMP2	*AM > intermedin > α-CGRP, AMY > sCT*
AM2	CLR + RAMP3	*AM ≥ intermedin ≥ α-CGRP > AMY > SCT*

Rank order of potency for endogenous peptide ligands for calcitonin receptor family of receptors. *sCT* Salmon CT, *hCT* Human CT, *AMY1* Amylin 1 receptor, *AMY2* Amylin 2 receptor, *AMY3* Amylin 3 receptor, *CGRP* Calcitonin gene related peptide receptor

Although CTR is most commonly known for its role in bone and calcium homeostasis (reviewed in [12]), its expression has been demonstrated in a number of cancer cell lines and primary cancers including breast and prostate cancers, bone cancers, leukemia, multiple myeloma, thymic lymphoma and glioblastoma (reviewed in [12]). Research on the role of CTR expression in cancer has been fragmentary and any role for CTR in cancer pathology seems to be entirely dependent on the cancer type. For instance, in human breast cancer model cell lines with high constitutive ERK (Extracellular Signal Regulated Kinase 1/2) phosphorylation, activation of CTR suppresses ERK phosphorylation. CT treatment inhibits the growth of MDA-MB-231 xenograft tumours but not those generated from MCF-7 cells [13]. In the human prostate cancer cell line PC3, CT inhibits apoptosis and stimulates tumour growth and invasiveness by recruiting zonula occludens-1 and promoting PKA-mediated tight junctions disassembly [14, 15]. Further, a metastatic derivative cell line – PC3M – expresses both CT and CTR and this co-expression appears to form a positive feedback system that increases invasiveness, emphasizing the role of paracrine/autocrine signaling of CT/CTR in this cancer [16, 17]. These data are also consistent with the European Medicines Agency report, recommending close monitoring of prostate cancer during the clinical use of CT (*EMA/109665/2013*). Furthermore, in mouse thymic lymphoma models, where CTR is expressed as an amylin receptor, amylin treatment leads to metabolic reprogramming (switch from glycolysis to oxidative phosphorylation) resulting in increased susceptibility to apoptosis [18, 19].

In normal human brain, CTR expression has been demonstrated by immunohistochemistry in the hypothalamus, limbic system and circumventricular organs in the brain stem [20] but not elsewhere, which is consistent with sites for radio-ligand binding and pharmacological effects in model animals (reviewed in [12]) and with data from the Human Protein Atlas [21]. Glioblastoma primary tumours are almost exclusively found in the cortex, being predominantly located in frontal and temporal lobes [22, 23], where CTR is not normally expressed. In glioblastoma biopsies, CTR expression has been detected using CTR-specific antibodies (12 out of 14 GBM biopsies were CTR positive), with low or undetectable CTR expression in adjacent non-tumour tissue [24]. This expression correlated with GBM stem cell morphology and co-expression of GBM stem cell markers, glial fibrillary acidic protein and CD133 [24]. Additionally, toxin conjugates of monoclonal anti-CTR antibody mAb2C4 promote cell death in JK2, SB2b and WK1 high-grade glioma and U87MG glioblastoma-derived cell lines with effective concentrations in the picomolar range, supportive of CTR expression [25]. Other recent reports show variable expression of CALCR mRNA with expression in only a subset 12/42 [26] or 115/152 [27] of primary tumours. In addition, Pal et al. [28] report a correlation between patient survival and non-synonymous mutations in CTR.

Other receptors of the CTR family were also found in GBMs. CALCRL/RAMP2 (Adrenomedullin 1 (AM1) receptor) and CALCRL/RAMP3 (Adrenomedullin 2 (AM2) receptor) mRNA has been detected in both human glioma biopsies and in GBM cell lines [29, 30]. Comparative expression of 138 different GPCRs has revealed high levels of CALCRL mRNA, specifically in human glioblastoma cancer stem-like cells compared to significantly lower expression in human brain tumour U87MG cells, human astrocytes and foetal neural stem cells [31], although CALCRL is widely expressed in normal brain [21] as it functions as the predominant receptor for the neuropeptide CGRP. It has also been shown that the degree of adrenomedullin peptide expression correlates with GBM tumour grade, with highest expression in grade IV tumours, where adrenomedullin is localised in proximity to large necrotic areas together with vascular endothelial growth factor (VEGF) [30, 32].

The existing data on the role of CTR in GBM are from correlative studies [24] and the study of Pal et al. [28] supports a model in which CTR is a tumour suppressor. There are currently no data that mechanistically address the role of CT/CTR in the progression of GBM. The widely available GBM model cell lines such as U87MG and A172 fail to recapitulate the original tumour in intracranial xenograft models and lose temozolomide resistance [33–35]. A new method of deriving GBM cell lines from single patient tumours has been established [36, 37]. These cell lines, grown in serum free media with the addition of defined growth factors, fully recapitulate the primary GBM tumour when injected into mice [38].

In this study we investigated further the question of whether CTR (or its family members) could be a valid therapeutic target in GBM treatment, as has been argued elsewhere [28]. For this purpose, we characterized expression of the calcitonin family of receptors along with the ligands, calcitonin and amylin (as they have been implicated in autocrine regulation of tumour growth) and performed functional and pharmacological characterisation across the known CTR signalling pathways.

## Methods

### Cell culture

Primary patient high-grade glioma (HGG) cell lines, in vitro surrogates of glioblastoma, were developed by QIMR-Berghofer Medical Research Institute (Brisbane, Australia) and represent 3 distinct GBM subtypes: SB2b and PB1 (classical), JK2 (proneural) and WK1(mesenchymal) [37], who supplied these directly for this study. GBM cell lines were cultured as adherent monolayers on matrigel (BD Biosciences)-coated flasks using StemPro NSC SFM serum free cell culture medium (Thermo Fisher Scientific) supplemented with 20 ng/ml EGF and 20 ng/ml FGFb, 1% D-glutamine and 1% of penicillin/ streptomycin (further referred as StemPro complete medium). Cells were cultured in 5% CO_2_ / 95% humidified air at 37 °C.

### cAMP (cyclic adenosine mono-phosphate) assay

GBM cell lines were seeded at 38000 cells/well (SB2b) or 35,000 cells/well (PB1, JK2 and WK1) in 96-well matrigel-coated plates and incubated for 24 h at 37°C, 5% CO_2_ in humidified incubator in StemPro complete medium. Media was replaced with stimulation buffer (phenol red free F12 media, 0.1% BSA, 0.5 mM IBMX, pH 7.4). Cells were stimulated with agonists (sCT, hCT, rAmy, CGRP or adrenomedullin) at concentrations ranging from 10^− 6^ M – 10^− 12^ M, or 10^− 5^ M forskolin, or vehicle for 30 min at 37 °C. Cells were lysed (0.3% Tween 20, 5 mM Hepes, 0.1% BSA, pH 7.4) and 5 μl of cell lysate from each well was transferred to a corresponding well of 384-well optiplate. Intracellular cAMP levels in the wells were determined using Lance Ultra cAMP assay kit (Perkin Elmer) according to the manufacturer’s instructions and detected using an Envision multilabel 2103 reader. Raw RFU values were converted using a cAMP standard curve to give absolute cAMP concentrations. Data were analysed by three-parameter logistic curve and are presented as percentage of 10^− 5^ M forskolin response.

### ERK 1/2 phosphorylation

SB2b cells were seeded at 38000 cells/well in 96-well matrigel-coated plates and incubated either overnight (for 4 h growth factor starvation), or for 7 h (for overnight starvation) at 37 °C, 5% CO2 in humidified incubator in StemPro complete medium. Culture media was replaced with DMEM/F12 GlutaMax medium (without growth factors) (Invitrogen, Carlsbad, CA, USA) and incubated for either 4 h (for 4 h growth factor starvation), or overnight (overnight starvation). An initial time-course was performed for each ligand (sCT and hCT) at 1 μM to assess the maximum peak of ERK_1/2_ phosphorylation. Following stimulation by ligands, media was removed and cells lysed in lysis buffer (TGR Bioscience). For ERK_1/2_ inhibition, test cells were stimulated with agonists (sCT and hCT, at concentrations ranging from 10^− 6^ M – 10^− 12^ M in presence of 0.3% FBS for 6 mins). ERK_1/2_ phosphorylation was detected using AlphaScreen SureFire pERK_1/2_ (Thr202/Tyr204) Assay Kit according to the manufacturer’s instructions and detected using an Envision multilabel 2103 reader.

### Ca^2+^ mobilization

GBM cell lines were seeded at 38000 cells/well (SB2b) or 35,000 cells/well (PB1 and WK1) in 96-well matrigel-coated plates and incubated for 24 h at 37 °C, 5% CO2 in humidified incubator in StemPro complete medium. Cells were washed twice with Ca^2+^ Buffer (150 mM NaCl, 10 mM HEPES, 10 mM D-glucose, 2.6 mM KCl, 1.18 mM MgCl_2_, 2.2 mM CaCl_2_, 0.5% BSA, 4 mM probenecid, pH 7.4) before addition of 1 μM Fluo4-AM diluted in Ca^2+^ buffer. Cells were incubated at 37°C for 60 min before ligand addition and detection of Ca^2+^ mobilisation in a FlexStation 3 (Molecular Devices). The machine settings were as follows: 37°C, excitation 485 nm, emission 525 nm, baseline reads of 15 s before drug addition, fast drug dispense, 120 s reading.

### Quantitative real-time reverse transcription polymerase chain reaction (qRT-PCR)

Cells were grown as indicated above in 6-well plates, rinsed in warm PBS, and plates rapidly frozen and stored at − 80 °C. Each n number refers to a different passage number of cells. Total RNA was extracted from 1 × 6-well plate using TriReagent (Sigma Aldrich, NSW, Australia) as per the manufacturer’s instructions. The yield and quality of RNA was assessed by measuring absorbance at 260 and 280 nm (Nanodrop ND-1000 Spectrophotometer; NanoDrop Technologies LLC, Wilmington DE USA) and by electrophoresis in 1.3% agarose gels. Any contaminating DNA was removed using the DNA-free DNA removal kit (Ambion, Thermo Fisher Scientific, Scoresby, Australia) as per manufacturer’s instructions. RNA samples were stored at − 80 °C. For preparation of cDNA, 0.5 μg of RNA was reverse-transcribed using iScript Reverse Transcription Supermix for RT-qPCR (Bio-Rad, Hercules, USA) according to the manufacturers instructions. Briefly, the reactions consisted of 2 μl of 5 x iScript reverse transcription supermix, 3 μl DNase/RNase free water, and 0.5 μg of RNA, in a final volume of 10 μl in 200 μl Eppendorf PCR tubes. Reactions were performed on a Applied Biosystems 2720 Thermal Cycler (Applied Biosystems, Foster City CA USA) as following: 25 °C for 5 min, 42 °C for 30 min, 85 °C for 5 min, and then allowed to cool to 4 °C. The cDNA was diluted with 190 μl DNase/RNase free water to obtain the equivalent of 2.5 ng/μl of starting RNA, and cDNA was stored at − 20 °C.

For each independent sample, qPCR was performed in duplicate using TaqMan Gene Expression assays (Life Technologies, MA, USA) for CALCR (Hs01016882_m1), CALCRL (Hs00907738_m1), RAMP1 (Hs00195288_m1), RAMP2 (Hs01006937_g1), RAMP3 (Hs00389131_m1), CALCA (Hs01100741_m1), IAPP (Hs00169095_m1), and ACTB (Hs01060665_g1). Each reaction consisted of 4 μl cDNA, 0.5 μl TaqMan Gene Expression Assay, 0.5 μl DNAse/RNase free water, and 5 μl TaqMan Fast Advanced Master Mix dispensed in Eppendorf twin.tec PCR plates. qPCR reactions were carried out using an Eppendorf Mastercycler ep Realplex Real-time PCR instrument. After initial heating at 50 °C for 2 min and denaturation at 95 °C for 10 min, fluorescence was detected over 40 cycles (95 °C for 15 s, 60 °C for 1 min). Data are expressed as relative expression of the gene of interest to the reference gene ACTB where:

$$ Relative\ expression={2}^{-\left(\left( Cq\  of\ gene\ of\ interest\right)-\left( Cq\  of\ ACTB\right)\right)} $$

For some genes, no mRNA was detected. These samples are omitted from the graphs as indicated in the figure legends.

### Western blotting

Cells were harvested by scraping into PBS containing 1x cOmplete Mini Protease Inhibitor Cocktail (Sigma-Aldrich) and 1 mM EDTA and subjected to small-scale subcellular protein fractionation to enrich for the membrane fraction. Briefly, cell suspensions were microfuged for 5 min at 350 g and 4 °C, supernatant discarded and cell pellet homogenised in 2.5 volumes of hypotonic buffer (10% glycerol, 10 mM pH 7.4 HEPES, 10 mM NaCl, 1.5 mM MgCl_2_) supplemented with 1 mM dithiothreitol (DTT; Sigma-Aldrich), 1 mM PMSF (Sigma-Aldrich) and 1x cOmplete Mini Protease Inhibitor Cocktail (Sigma-Aldrich) and incubated for 30 min on ice followed by centrifugation for 30 min at 16,300 g and 4 °C. The supernatant was discarded and the cell pellet resuspended and homogenised in hypertonic buffer (10% glycerol, 420 mM NaCl, 10 mM pH 7.4 HEPES, 1.5 mM MgCl_2_) with 1 mM DTT, 1 mM PMSF and 1x cOmplete Mini Protease Inhibitor Cocktail followed by 30 min on ice and centrifugation for 30 min at 16,300 g and 4 °C. The supernatant was discarded and the cell pellet resuspended and homogenised in radio immunoprecipitation (RIPA) buffer [1% NP-40 (Sigma-Aldrich), 1% sodium deoxycholate, 0.1% SDS, 150 mM NaCl, 50 mM NaF, 10 mM pH 7.2 phosphate buffer, 0.2 mM EDTA] and with 1 mM DTT, 1 mM PMSF and 1x cOmplete Mini Protease Inhibitor Cocktail. Suspensions were kept on ice for 10 min to allow solubilisation of membrane proteins and centrifuged for 20 min at 16,300 g and 4 °C. The resulting supernatant (membrane protein fraction) was collected and all fractions stored at − 80 °C. 50 μg of BCA quantitated membrane fractions were denatured at room temperature in Laemmli sample buffer and subjected to electrophoresis on 8% gels and transfer to PVDF using standard methods. Membranes were probed with anti-CTR antibody (1H10 Welcome Receptor Antibodies, Melbourne, Australia; MCA2191, BioRad AbD Serotec, Kidlington, UK) followed by HRP-conjugated secondary, enhanced chemi-lumiscence and detection with a LAS-3000 Imaging System (Fuji; Tokyo, Japan).

### Public data analysis

Raw data from IVY-GAP was analysed as follows: patient tumour biopsies were manually curated to identify tumour blocks with RNA-seq expression of CALCR greater than 1.25 FPKM that also corresponded to histological grading consistent with GBM, resulting in the identification of 12 patients whose tumours were positive for CALCR expression. This threshold was then applied to the patient survival data to generate a Kaplan-Meier survival plot. Raw RNA-seq data from TCGA was used to identify tumour samples with CALCR gene expression, this data was used to filter the patient clinical data and expression was converted to Log_2_. This was used to generate an expression – survivorship plot shown in Additional file 1: Figure S2. This data was used with an FPKM threshold of 1.25 to generate the Kaplan-Meier plot shown in Fig. 5d.

## Results

Data from the Q-Cell database [37] for glioblastoma cell lines are reproduced in Table 2 showing key meta-data relating to donor age and survival for the cell lines examined.

**Table 2:** Key patient meta-data and derived cell line properties (adapted from Q-Cell database [35])

Cell line	Patient age (years)	Patient Survival (Days)	RB pathway LOF	Cell line doubling time (hours)	XG median survival (days)
BAH1	75	94	Yes	79.5 ± 3.3	210 ± 8
FPW1	68	242	Yes	48.1 ± 4.7	196 ± 4
HW1	54	89	Yes	55.8 ± 2.7	174 ± 14
JK2	75	178	Yes	94.2 ± 4.5	147 ± 9
MMK1	80	334	Yes	52.4 ± 3.3	157 ± 15
MN1	84	36	Yes	44.9 ± 1.0	258 ± 20
PB1	57	39	Yes	79.4 ± 6.3	71 ± 1
RKI1	57	Alive (7 years)	No	72.9 ± 5.3	248 ± 6
RN1	56	243	Yes	37.5 ± 1.9	81 ± 2
SB2b	48	420	Yes	108.7 ± 6.9	120 ± 3.3
SJH1	72	45	Yes	67.3 ± 4.7	148 ± 4
WK1	77	121	Yes	46.2 ± 1.0	150 ± 2

Metadata for patient age and survival post-diagnosis along with retinoblastoma status, doubling time for the derived cell lines and median survival of mice carrying orthotopic tumours. *RB pathway LOF* Loss of function in one or more steps in the retinoblastoma tumour suppressor pathway. *XG median survival* Median survival for mice with orthotopic xenografts

**Fig. 1:**
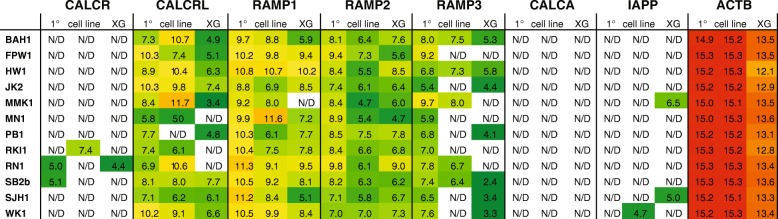
Microarray data for expression of calcitonin receptor family and selected calcitonin receptor family ligands. Log_2_ expression of calcitonin receptor family mRNA from Illumina micro array on primary tumour biopsies (1°), decived cell lines and orthotopic xenographs (XG). Expression is intensity colour coded from green (lowest) through yellow (middle) to red (highest). N/D represents microarray data for which there was either no signal detected or the detection *P*-value fell above 0.05

**Fig. 2:**
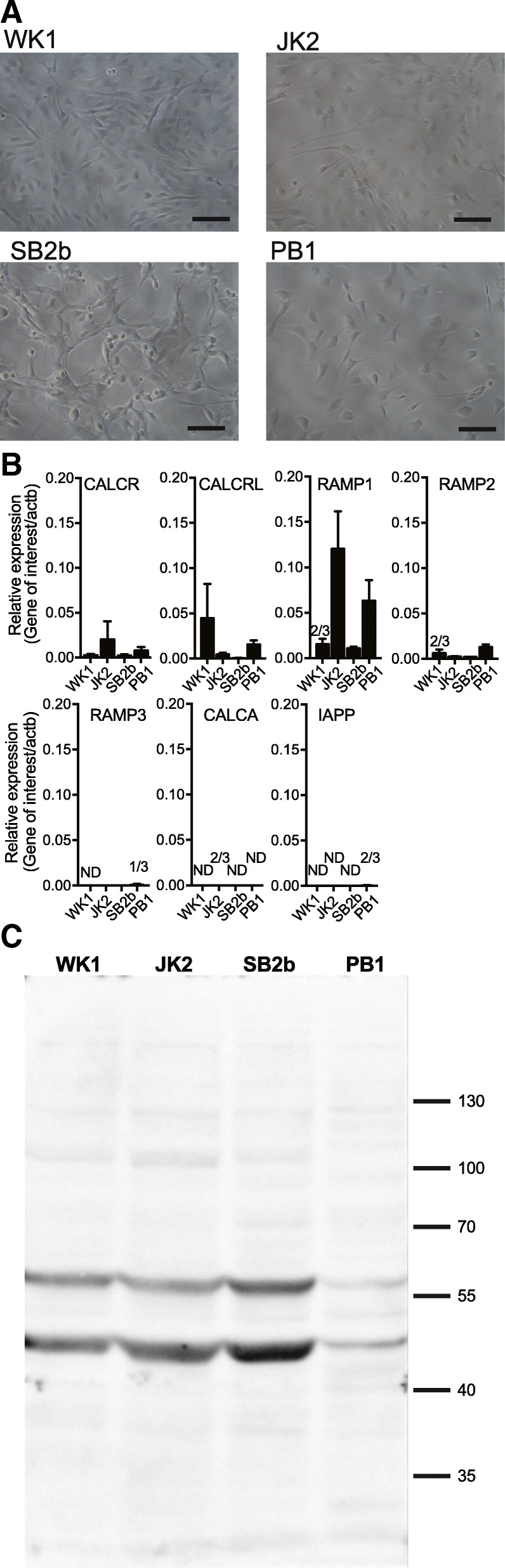
Morphology and gene expression in the four GBM cell lines used in this study. **a** SB2b and PB1 of classical GBM subtype; JK2 of proneural subtype and WK1 of mesenchymal GBM subtype as adherent cultures on matrigel, scale bar represents 100 μM. **b** Expression of CALCR, CALCRL, RAMP1, RAMP2, RAMP3, CALCA, and IAPP in SB2b, PB1, WK1 or JK2 cells. Data represent mean + SEM of 3 independent experiments, performed in duplicate, relative to β-actin expression. ND (not detected in all 3 samples), 2/3 (in 2 out of the 3 samples mRNA was detected), 1/3 (in 1 out of the 3 samples mRNA was detected). **c** Western blot for CTR, 50 μg of membrane protein probed with anti-CTR 1H10, representative of 3 independent experiments

We analysed our primary microarray data (normalised using Illumina BeadStudio) for expression in primary tumours, cell lines and xenograft tumours of calcitonin receptor family genes along with calcitonin and amylin by considering all microarray data with a detection *P*-value of < 0.05 (Fig. 1). In contrast to our previous data [24] and data available in public glioblastoma data bases (IVY-GAP [26] and TCGA [27]), CTR mRNA was only detected in 2/12 primary tumours, a single cell line and one xenograft (Fig. 1), possibly due to limitations in this approach as previously reported for GPCRs [39]. The CALCA message, which encodes both calcitonin and α-CGRP, was not detected in any sample and the mRNA encoding amylin (IAPP) was only detected in one cell line and 2 xenografts, none of which were concordant with detection of CALCR (Fig. 1). In contrast, but consistent with public databases, all primary tumours had detectable message for CALCRL and all RAMPs with one cell line losing detectable CALCRL expression along with 3 xenografts, one xenograft losing RAMP2 and 7 cell lines and 5 xenografts having no detectable RAMP3 (Fig. 1). We therefore performed a preliminary screen by western blot for CALCR in our 4 HGG cell lines, which supported expressed protein of molecular weight and reactivity consistent with CTR in all 4 cell lines (see below). From this preliminary experiment we chose to further characterise these 4 cell lines, which appeared to have detectable CTR and were previously used in an anti-CTR immunotoxin study [25]. These cells were grown as adherent cultures in a monolayer on matrigel with EGF and FGFb growth factors that, in the absence of serum, showed a consistent morphology to previous publications (Fig. 2a).

### CALCR, CALCRL and RAMPs are expressed in each of four GBM cell lines

Due to the discrepancy between the microarray data and our initial western blot screen, cell lines WK1, JK2, SB2b and PB1 were selected for further analysis. We tested for mRNA expression using TaqMan qPCR, which has been reported as more reliable for detection of low expression GPCR transcripts [39]. This showed low level of expression of both CALCR and CALCRL mRNA in all of the 4 cell lines (Fig. 2b). The apparent discordance between mRNA level and western blotting suggests that CTR protein level is primarily regulated at the translational or post-translational level as has been widely reported for non-housekeeping genes, where the concordance between mRNA and protein levels is low [40–42]. CALCR mRNA expression was markedly lower than CALCRL, at levels that are consistent with low mRNA copy number that is commonly seen for GPCRs and is also consistent with the low FPKM values extracted from the IVYGAP [26] and TCGA databases [27] (below). RAMPs 1 and 2 mRNAs were expressed in all 4 cell lines (Fig. 2b). RAMP3 was not detected in the SB2b cell line but was present at levels just above the threshold in PB1 and WK1 (approximately one copy per cell), and at slightly higher level in JK2. Expression of CALCA (encoding calcitonin and α-CGRP) and IAPP (encoding amylin) mRNA were also assessed, neither of which were detectable, except for very low (below the threshold of 1 copy per cell) IAPP in PB1 and CALCA in JK2 (Fig. 2b). We were most interested in expression of CALCR based on previous studies described above and therefore performed western blots using an anti-CTR Ab on purified membranes; these revealed antibody reactive bands corresponding to the expected size for unmodified (~ 50 kDa) and glycosylated (~ 60 kDa) CTR in all cell lines (Fig. 2c).

### Individual HGG cell lines have distinct CTR/CLR-based pharmacology

**Fig. 3:**
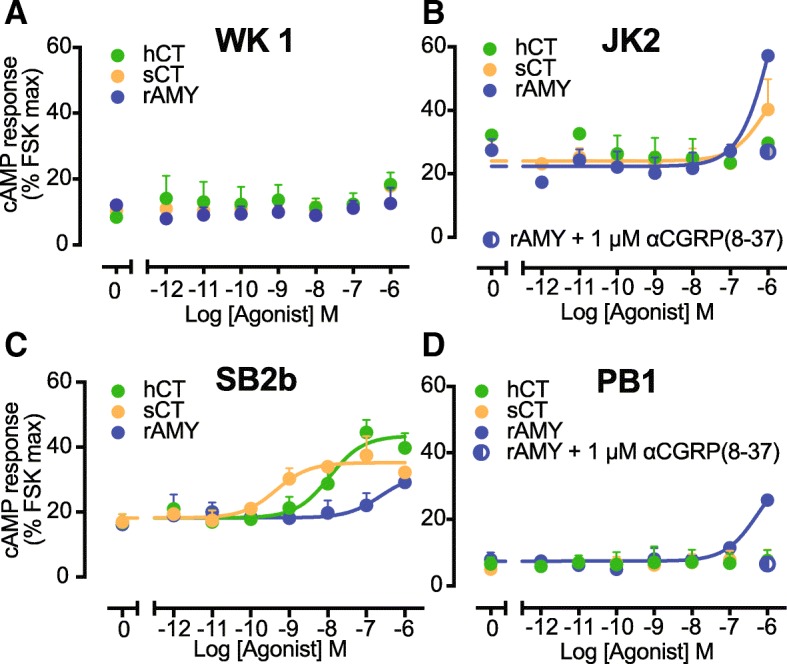
cAMP Signalling by CTR activated by its cognate agonists. Characterization of cAMP accumulation (30 min) in response to stimulation by sCT, hCT and amylin (rAmy) in WK1 (**a**), JK2 (**b**), SB2b (**c**) and PB1 (**d**) cell lines. Data are analysed using a three-parameter logistic curve. All values are mean + S.E.M. of 3 to 5 independent experiments conducted in triplicate

In a wide variety of recombinant and ex vivo settings CTR is most strongly coupled to the stimulatory hetero-trimeric Gα subunit, Gαs, that activates adenylate cyclase [43–47]. CTR function was therefore assessed using a cAMP accumulation assay. In the classical GBM model cell line, SB2b, we observed a robust, concentration dependent increase in cAMP in response to CTR agonists (Fig. 3c). The potency of the response to sCT (pEC_50_ = 9.4 ± 0.3) and hCT (pEC_50_ = 8.0 ± 0.2) were similar to the known affinities of these agonists for CTR. The similarities in affinity and cellular potency are consistent with low receptor expression and limited receptor reserve. We also observed a higher E_max_ for hCT (43% of forskolin) compared with sCT (35% of forskolin), consistent with higher efficacy of this lower affinity agonist [48]. The low potency amylin response (pEC_50_ = 6.8 ± 0.5) is consistent with signalling via CTR in the absence of any RAMP [49] indicating there is unlikely to be functional interaction between CTR and RAMPs in this cell line.

No pharmacologically relevant responses to either sCT or hCT, in cAMP assays were detected in the other 3 cell lines (PB1, JK2 and WK1) (Fig. 3a, b & e). In the mesenchymal model line, WK1, we observed a small increase in cAMP in response to maximal concentrations of all 3 CTR agonists (Fig. 3a). To address whether this was due to poor receptor coupling to adenylate cyclase we performed co-stimulation experiments in the presence of a sub saturating concentration of forskolin (1 μM). Application of 1 μM forskolin increased the basal cAMP 4-fold and revealed a low potency sCT response with a pEC50 of 6.7 ± 0.6 (Additional file 2: Figure S1A) that is inconsistent with a CTR-mediated response. The low potency response to amylin in JK2 (pEC_50_ = 5.9 ± 0.5) and PB1 (pEC_50_ = 6.1 ± 0.5) is inconsistent with an AMY receptor phenotype but is consistent with the CGRP (CLR/RAMP1) receptor, with application of 1 μM of the CGRP antagonist (CGRP(8–37)) abolishing this response (Fig. 3b, d).

In addition to coupling to Gαs, the CTR can couple to Gαq, which stimulates phospholipase C leading to intracellular calcium mobilisation (_i_Ca^2+^) [46]. We were unable to detect functional CTR response in an _i_Ca^2+^ mobilisation assay with sCT or amylin agonists in SB2b, WK1 or PB1 cell lines (Additional file 2: Figure S1B, C & D).

**Fig. 4:**
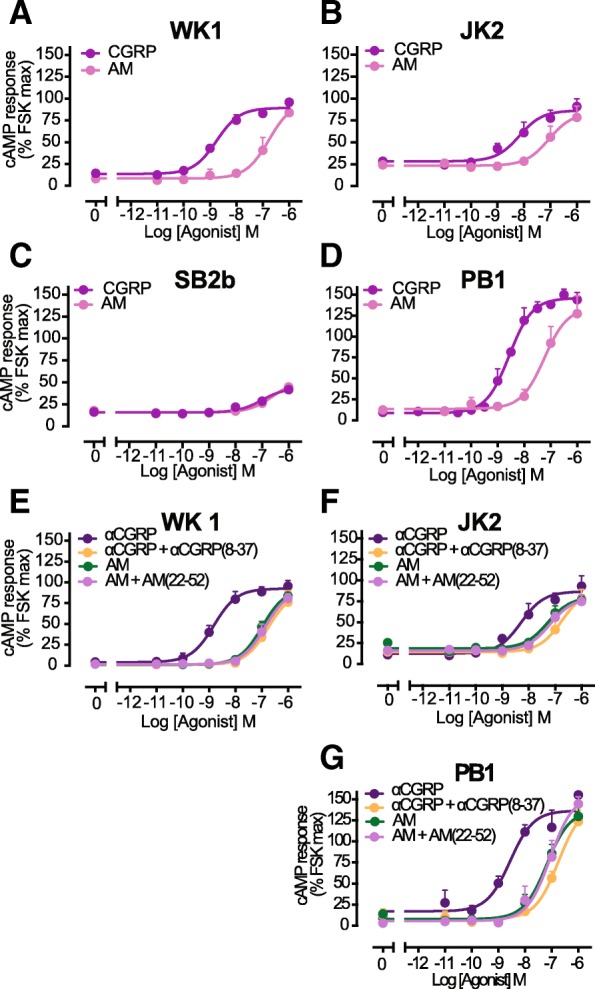
cAMP Signalling signalling by CLR activated by its cognate agonists & receptor subtype can be discriminated using specific antagoninsts. Characterization of cAMP accumulation (30 min) in response to stimulation by αCGRP and AM in WK1 (**a**), JK2 (**b**), SB2b (**c**) and PB1 (**d**) cell lines. Data are analysed by three-parameter logistic curve. All values are mean + S.E.M. of 3 to 4 independent experiments conducted in triplicate. Receptor discrimination in cAMP accumulation (30 min) in response to stimulation by either αCGRP and AM alone, or in presence of the antagonists αCGRP(8–37) or AM(22–52) in WK1 (**e**), JK2 (**f**) and PB1 (**g**) cell lines. Data were fit using a three-parameter logistic curve. All values are mean + S.E.M. of 3 to 4 independent experiments conducted in triplicate

Quantitation of mRNA indicated that all cell lines expressed CLR and at least one RAMP family member. We therefore performed cAMP accumulation assays in the presence of adrenomedullin and αCGRP. No pharmacologically relevant response was seen in the SB2b cell line (Fig. 4c) suggesting that no functional CLR is present at the cell surface. The remaining three cell lines (PB1, JK2 and WK1) responded with high potency to stimulation with αCGRP (pEC_50_ = 8.6 ± 0.1 for PB1, pEC_50_ = 8.3 ± 0.2 for JK2 and pEC_50_ = 8.8 ± 0.1 for WK1) and with lower potency to adrenomedullin (pEC_50_ = 7.3 ± 0.1 for PB1, pEC_50_ = 7.0 ± 0.3 for JK2 and pEC_50_ = 6.8 ± 0.3 for WK1) (Fig. 4a, b and d). These data are most consistent with the reported pharmacology of a CLR/RAMP1 CGRP receptor phenotype. CLR generates 3, pharmacologically distinct receptor subtypes depending on its interaction with the 3 different RAMPs. To distinguish receptor subtypes present we measured cAMP concentration response to αCGRP and AM in the presence of 1 μM subtype selective antagonists – αCGRP(8–37) and AM(22–52). Co-treatment of PB1, JK2 and WK1 cell lines with αCGRP and its specific antagonist, αCGRP(8–37) led to an approximate 100-fold decrease in potency of αCGRP response reflected as 2 logarithmic unit shift in the EC_50_ (pEC_50_ from 8.6 to 6.7 in PB1; from 8.4 to 6.6 in JK2; and from 8.8 to 6.6 in WK1) (Fig. 4e, f and g). In contrast, co-treatment with AM and its specific antagonist, AM:22–52 caused no significant shift in potency of AM response (pEC50 of 7.2 versus 7.1 in PB1; 7.2 versus 7.3 in JK2; and 6.9 versus 6.7 in WK1). This is consistent with PB1, JK2 and WK1 cell lines expressing CGRP and not AM receptors, with the weak AM responses, mediated through the CGRP receptor.

### Activation of CTR expressed in the SB2b cell line has no detectable effect on cell metabolism, proliferation, ERK phosphorylation or p38 phosphorylation

**Fig. 5:**
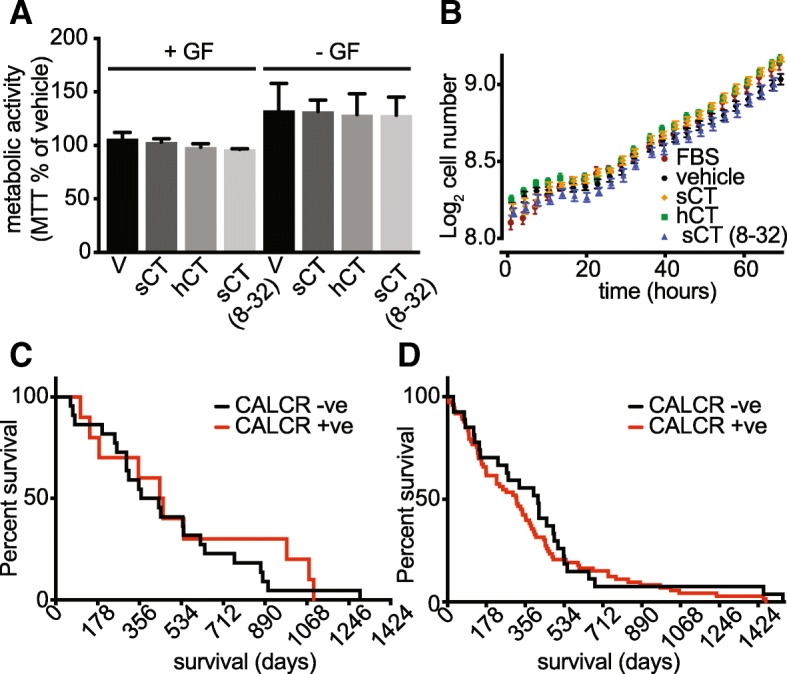
The role of CTR in metabolism, proliferation and patient outcome. **a** metabolic activity of SB2b was assessed by an MTT assay in the presence (+GF) or absence (−GF) of defined growth factors (EGF + bFGF) in combination with 1 μM of CTR agonists sCT or hCT or the CTR specific antagonist sCT(8–32), results are mean + S.E.M. of 3 independent experiments performed in triplicate. In **b**, the effect of 1 μM CTR ligands on cell proliferation was assessed using live cell imaging (Operetta) over 72 h, cell number per field is expressed as a Log_2_ value and for clarity only every 3rd data point is shown, with representative experiment (of 3 independent experiments). Kaplan-Meier plots were derived from publicly available data using a threshold for expression of 1.25 FKPM (~ 1 transcript per cell) for all patients for whom RNAseq and survival data was available, CALCR +ve means over 1.25 FKPM, CALCR -ve means less than 1.25 FKPM, in **c** is the data from the IVY-GAP database and **d** shows data from TCGA

Activation of the CTR, as part of the amylin receptor, has been shown to cause metabolic reprogramming in some malignancies [18]. Others [28] have reported a change in GBM cell line proliferation in response to CTR stimulation and this has also been reported for a breast cancer cell line [50, 51], we therefore assessed whether activation (using both hCT and sCT) or blockade (using the antagonist sCT(8–32)) would alter metabolism in the SB2b cell line. Although growth factor deprivation had a significant effect on cellular metabolism (as assessed by an MTT assay, Fig. 5a) we saw no effect of any of the CTR ligands (Fig. 5a). In addition, we observed no changes in cell proliferation in the SB2b cell line by live cell imaging in the presence of CTR ligands (Fig. 5b) over 3 days, relative to the unstimulated control. To assess more proximal effects of CTR activation on pathways involved in cell proliferation, we directly assessed the ability of sCT to activate pERK and p38 MAPKs, that have been implicated in tumour progression. We performed ERK_1/2_ and p38 phosphorylation assays as a time course in the SB2b cell line and were unable to detect any response (Additional file 1: Figure S2A and B). In some tumour cell lines, such as the glioblastoma model A172 [24] and the MDA-MB-231 breast cancer model [13], CTR stimulation is inhibitory for ERK_1/2_ phosphorylation. We therefore tested for pERK_1/2_ suppression by either sCT or hCT in the presence of 0.1% FBS. We saw no detectable inhibition by either agonist over the concentrations tested (Additional file 1: Figure S2C) and conclude that CTR does not modulate pERK_1/2_ within the sensitivity we are able to measure.

### CTR expression and survivorship

To understand what (if any) relationship CTR expression may have with tumour progression and patient outcome we extracted the raw expression data for CTR from two public databases (IVY-GAP [26] and TCGA [27]). We set an arbitrary cut-off for CTR expression corresponding to ~ 1 transcript per cell (Log_2_ FPKM = 0.3) [52]. For patients for whom survival data were available, we used this threshold to generate Kaplan-Meier plots (Fig. 5c and d). In the IVY-GAP data, using our threshold, 12 out of 42 patient tumours (~ 28%) were positive for CTR and there was no relationship between CTR expression and survivorship (Fig. 5c). In the TCGA dataset, 115 of 152 patient tumours were CTR positive (~ 76%) by our criteria and similarly to the IVY-GAP dataset, there was also no relationship between CTR expression and survivorship (Fig. 5d); this result that was borne out when we plotted expression against survivorship for the TCGA dataset (Additional file 1: Figure S2D). Pal et al. [28] reported that several mutations of CTR were associated with loss-of-function (LOF) that correlated with poor patient outcome. To investigate the possibility that the lack of signalling of CTR that we report might be related to loss-of-function mutations we analysed whole exome sequencing data. The exome sequencing data showed that each of the cell lines bear a known polymorphism encoding leucine in the c-terminal tail of the protein (NM_001164737:c.T49C:p.S17P; JK2 (heterozygous), PB1 (homozygous), SB2b (homozygous) and WK1 (heterozygous)) that has a minor consequence on signalling [53]. The only other identified non-synonymous polymorphism was identified in the WK1 cell line (NM_001164738:c.G1369A:p.E457K, heterozygous), which would be predicted to result in a glutamine to lysine change in the distal C-terminus, an amino acid that is substituted with glycine, leucine and valine amongst mammals (Additional file 3: Figure S3) and a part of the c-terminus that can be deleted without altering cAMP coupling [54]. To contextualise the reported LOF mutations of Pal et al. [28], we mapped these to our current model of active CT bound CTR [54] (Additional file 3: Figure S3) and compared these residues across vertebrate species (Additional file 4: Figure S4). As shown in Additional file 3: Figure S3, our model would only predict that P100L and R404C may effect receptor signalling and these mutations show the least LOF as reported by Pal et al. [28].

## Discussion

Expression profiles revealed by RT qPCR indicate that each of the four model HGG cell lines investigated here generate mRNA encoding components of CTR, AMY, CGRP and AM_1_ receptors, albeit that the CTR was expressed at low levels. Neither of the CTR activating peptides, hCT or amylin, were expressed at levels above threshold, suggesting there is no autocrine production of these peptides by these GBM cell lines. No coding polymorphisms, apart from the well-characterised c-terminal tail leucine/proline polymorphism [53], were detected by whole exome sequencing.

Although, RT qPCR data show mRNA encoding multiple receptors of the CTR family in the SB2b cell line (classical GBM subtype), functionally we were only able to confirm CTR. The potency with which CTR agonists elicit a cAMP response is consistent with a low level of endogenously expressed receptor. Weak responses to αCGRP, amylin and adrenomedullin can be attributed to CTR activation. In this cell line, sCT and hCT had distinct profiles with sCT demonstrating higher potency but lower E_max_ than hCT. The molecular basis for the signalling profile observed in the SB2b GBM cell line is consistent with low or no receptor reserve but reveals apparent differences in efficacy, consistent with our data in recombinant systems [48], and further illustrates the complexity of signaling in (patho)physiologically relevant systems. In our assays we couldn’t detect a response to sCT in _i_Ca^2+^ mobilization assay suggesting limited Gq coupling of CTR in the SB2b cell line. Additionally, we were not able to detect either pERK_1/2_ or p38 MAPK response in this cell consistent with no downstream effect of CTR activation on cellular metabolism or proliferation.

PB1, JK2 and WK1 cell lines (representing classical, proneural and mesenchymal types of GBM respectively) had detectable mRNA and western blot immuno-reactivity consistent with CTR expression (C-terminally directed antibody). In spite of this we could not detect a functional CTR response as assessed by cAMP accumulation (PB1, JK2, WK1) and _i_Ca^2+^ assay (PB1 and WK1). However, we have previously shown that both JK2 and WK1 cell lines are susceptible to anti-CTR-immunotoxin (N-terminally directed) mediated cell killing [25]. In other systems, CTR has been reported to internalise extremely rapid in a ligand independent receptor manner [53, 55], suggesting that perhaps the CTR may not be present at the plasma membrane for sufficient time to generate detectable functional response in these GBM lines.

Despite a lack of functional CTR in PB1, JK2 and WK1 cell-lines, all three displayed a potent cAMP accumulation response to αCGRP, a less potent response to adrenomedullin, with a very weak response to amylin. In addition, specific CGRP and AM receptor antagonists confirmed the presence of CLR/RAMP1 type CGRP and not an AM receptor in these cells. This is in agreement with RT qPCR gene expression data showing expression of both CLR and RAMP1. However, CGRP is unlikely to be tractable as a target for treating GBM given its broad expression in the brain. There was no clear correlation between pharmacological profiles and clinical classification of the originating GBM tumour subtype as SB2b and PB1, both of classical GBM subtype, had distinct pharmacological profiles of CTR and CLR/RAMP1 receptor.

## Conclusion

Taken together this data indicates that we have a rather incomplete understanding of CTR (and related receptor) function in certain (patho)-physiological circumstances. While the CTR is expressed in a significant subset of GBM tumours it may only be tractable as a target by leveraging the compromised blood brain barrier characteristic of these tumours while taking advantage of the rapid cycling of CTR to deliver a toxic payload.

Our analysis of published CTR expression from IVY-GAP [26] or TCGA [27] databases do not support a correlation between CTR expression and patient outcome. We would therefore argue that CTR expression, while common in primary GBM tumours, is unlikely to be tractable to pharmacological intervention but may be suitable as a target for delivering cytotoxic agents.

## Additional files

Additional file 1: **Figure S2.** MAP kinase response to sCT in SB2b cells and TCGA survival data. No detectable ERK_1/2_ phosphorylation (A) or p38 (B) in response to stimulation with 1 μM sCT in SB2b cell line while a robust response to 10% FBS is seen; Data are presented as mean + S.E.M. of 3 replicates of a representative experiment. (**C**) ERK_1/2_ Phosphorylation response in SB2b cell line was induced by 0.1% FBS. No suppression of the induced response after stimulation sCT or hCT was seen at the concentrations tested (C). Data are presented as mean + S.E.M. of 3 replicates of a representative experiment. (D) Log_2_ expression (FPKM) ofr CALCR transcript in patients with survival data from the TCGA database plotted as a scatter plot against survival. (PDF 915 kb)

Additional file 2: **Figure S1.** cAMP and _i_Ca^2+^ mobilization in response to CTR agonists. A, Characterization of cAMP accumulation (30 min) in WK1 cells in response to stimulation by sCT alone or in presence of 1 μM forskolin. Data are presented as mean + S.E.M. of 3 replicates of a representative experiment. Absence of intracellular calcium mobilization response to sCT and rAMY in WK1 (B), SB2b (C) and PB1(D) cell lines while maintaining robust response to 10 μM ATP and 1 μM ionomycin. Data are presented as peak values of response measured in relative fluorescence units. Data are presented as mean + or - S.E.M. of 3 replicates of a representative experiment. (PDF 907 kb)

Additional file 3: **Figure S3.** Mapping reported CTR mutations to our a molecular model of the CTR [48]. A, mutations reported to be associated with LOF at the CTR are shown in space fill red, mapped onto our active, G protein bound, model derived from Cryo-EM data,; the peptide (sCT) is shown in orange, receptor in blue, Gα subunit in yellow, Gβ in teal and Gγ in purple. B, the reported LOF residues, their substitution, mammalian conservation structural location, potential side-chain interaction and likely effect on receptor function are shown as a table. (PDF 3120 kb)

Additional file 4: **Figure S4.** Alignment of vertebrate CTR sequences. Alignment of a subset of validated and predicted CTR sequences from mammals and aves with reptile and amphibian sequences used as outgroups. Sequences were obtained from NCBI homologene filtering for reference sequences only. These were then manually curated and an alignment was performed using Clustalw Omega. Conserved asparagine (yellow) and cysteine (purple) residues in the N-terminus have been manually annotated and TMMHM used to predict TM helices which were manually curated and are indicated in blue. Putative LOF mutations are highlighted in red. (PDF 211 kb)

## Abbreviations

ACTB: Actin b (gene)
AM: Adrenomedullin
Amy: Amylin
BCA: Bicinchoninic acid assay
CALCA: Calcitonin related polypeptide alpha (gene)
CALCR: Calcitonin receptor (gene)
CALCRL: Calcitonin-like receptor (gene)
cAMP: 3′, 5′-cyclic adenosine monophosphate
CGRP: Calcitonin gene related peptide (peptide)
CLR: Calcitonin-like receptor (protein)
cDNA: Complimentary deoxyribose nucleic acid
CT: Calcitonin (peptide)
CTR: Calcitonin receptor (protein)
DMEM: Dulbecco’s modified Eagle medium
DNA: Deoxy-ribose nucleic acid
DNase: Doexyribse nuclease
EGF: Epidermal growth factor
ERK_1/2_: Extracellular signal-regulated kinase 1 and 2
FBS: Fetal bovine serum
FGFb: Basic fibroblast growth factor
FPKM: Fragments Per Kilobase of transcript per Million mapped reads
GBM: Glioblastoma
GLP-1: Glucagon-like peptide-1
GPCR: G protein-coupled receptor
HGG: High-grade glioma
HRP: Horse radish peroxidase
IAPP: Islet amyloid polypeptide (gene)
IBMX: 3-Isobutyl-1-methylxanthine
_i_Ca^2+^: Intracellular Calcium
IVY-GAP: Ivy Glioblastoma Atlas Project
LOF: Loss of function
mRNA: Messenger ribonucleic acid
NSC: Neural stem cell
PBS: Phosphate buffered saline
PCR: Polymerase chain reaction
PKA: Protein kinase A
PVDF: Polyvinylidene difluoride
RAMP: Receptor activity modifying protein
RNA: Ribonucleic acid
RNase: Ribonuclease
RT-qPCR: Reverse-transcription quantitative polymerase chain reaction
SFM: Serum-free media
TCGA: The Cancer Genome Atlas
VEGF: Vascular endothelial growth factor
