# Supplementary figures and images for: Expression and activity of the calcitonin receptor family in a sample of primary human high-grade gliomas
Source: BMC Cancer. 2019 Feb 18;19:157. doi: 10.1186/s12885-019-5369-y (PMC6379965; doi:10.1186/s12885-019-5369-y)

# Supplementary Figure 2

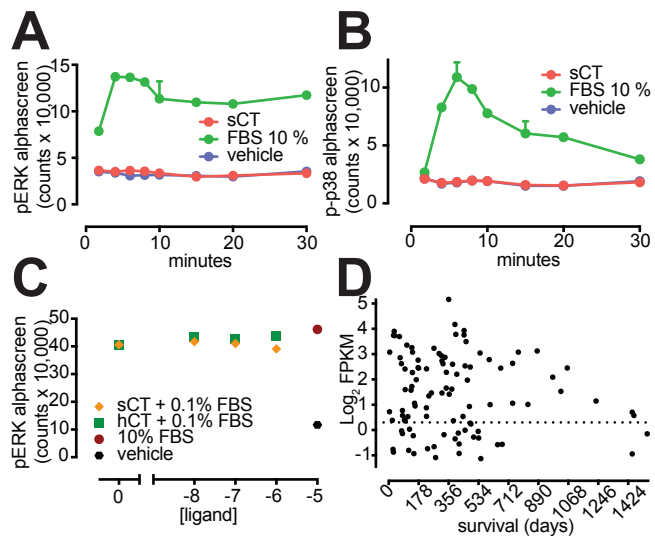

Supplement: Supplementary file 1 — Figure S2. MAP kinase response to sCT in SB2b cells and TCGA survival data. No detectable ERK1/2 phosphorylation (A) or p38 (B) in response to stimulation with 1 μM sCT in SB2b cell line while a robust response to 10% FBS is seen; Data are presented as mean + S.E.M. of 3 replicates of a representative experiment. (C) ERK1/2 Phosphorylation response in SB2b cell line was induced by 0.1% FBS. No suppression of the induced response after stimulation sCT or hCT was seen at the concentrations tested (C). Data are presented as mean + S.E.M. of 3 replicates of a representative experiment. (D) Log2 expression (FPKM) ofr CALCR transcript in patients with survival data from the TCGA database plotted as a scatter plot against survival. (PDF 915 kb) [file 12885_2019_5369_MOESM1_ESM.pdf]

# Supplementary Figure 1

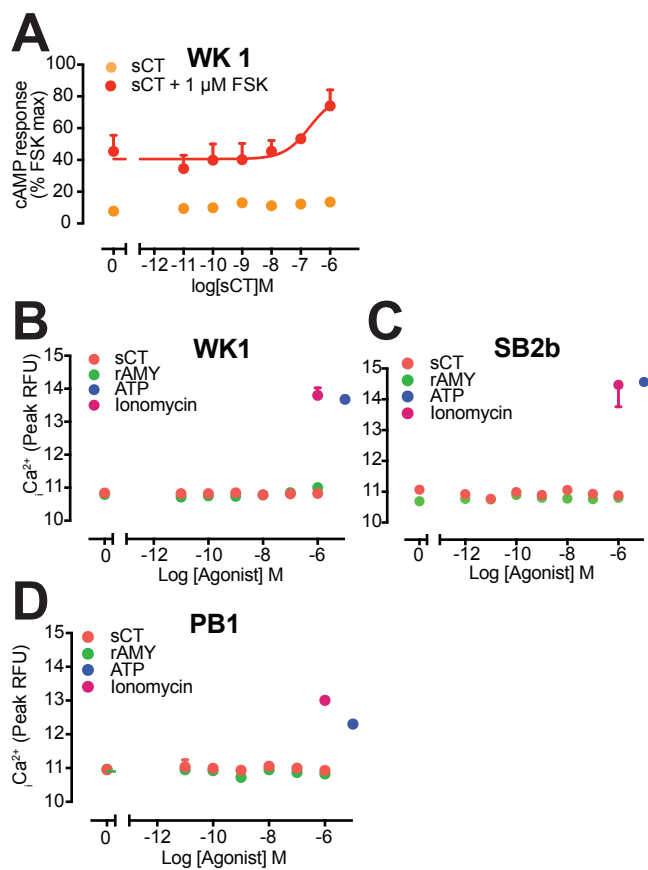

Supplement: Supplementary file 2 — Figure S1. cAMP and iCa2+ mobilization in response to CTR agonists. A, Characterization of cAMP accumulation (30 min) in WK1 cells in response to stimulation by sCT alone or in presence of 1 μM forskolin. Data are presented as mean + S.E.M. of 3 replicates of a representative experiment. Absence of intracellular calcium mobilization response to sCT and rAMY in WK1 (B), SB2b (C) and PB1(D) cell lines while maintaining robust response to 10 μM ATP and 1 μM ionomycin. Data are presented as peak values of response measured in relative fluorescence units. Data are presented as mean + or - S.E.M. of 3 replicates of a representative experiment. (PDF 907 kb) [file 12885_2019_5369_MOESM2_ESM.pdf]
