# Supplementary material for: Expression and activity of the calcitonin receptor family in a sample of primary human high-grade gliomas
Source: BMC Cancer. 2019 Feb 18;19:157. doi: 10.1186/s12885-019-5369-y (PMC6379965; doi:10.1186/s12885-019-5369-y)
Supplement: Supplementary file 3 — Figure S3. Mapping reported CTR mutations to our a molecular model of the CTR [48]. A, mutations reported to be associated with LOF at the CTR are shown in space fill red, mapped onto our active, G protein bound, model derived from Cryo-EM data,; the peptide (sCT) is shown in orange, receptor in blue, Gα subunit in yellow, Gβ in teal and Gγ in purple. B, the reported LOF residues, their substitution, mammalian conservation structural location, potential side-chain interaction and likely effect on receptor function are shown as a table. (PDF 3120 kb) [file 12885_2019_5369_MOESM3_ESM.pdf]

Supplementary Figure 3

A

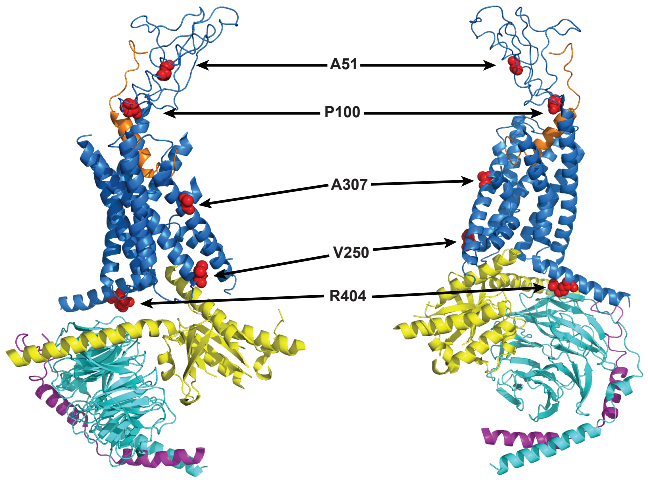

B

Residue	GBM Substitution	Vertebrate Substitutions	Location	Side chain	Likely impact
**R45**	Q	K,R	Unresolved (NTD)	Unresolved	none
**A51**	T	S	NTD	solvent	none
**P100**	L	Q	NTD	Toward ligand	Altered ligand binding
**V250**	M	M	TMIII	Lipid bilayer	none
**A307**	V	V	TMV	Lipid bilayer	none
**R404**	C	conserved	CTD	Gβ interaction	Effector coupling
**R420**	C	H	Unresolved (CTD)	Unresolved	none
