# Supplementary material for: Expression and activity of the calcitonin receptor family in a sample of primary human high-grade gliomas
Source: BMC Cancer. 2019 Feb 18;19:157. doi: 10.1186/s12885-019-5369-y (PMC6379965; doi:10.1186/s12885-019-5369-y)
Supplement: Supplementary file 4 — Figure S4. Alignment of vertebrate CTR sequences. Alignment of a subset of validated and predicted CTR sequences from mammals and aves with reptile and amphibian sequences used as outgroups. Sequences were obtained from NCBI homologene filtering for reference sequences only. These were then manually curated and an alignment was performed using Clustalw Omega. Conserved asparagine (yellow) and cysteine (purple) residues in the N-terminus have been manually annotated and TMMHM used to predict TM helices which were manually curated and are indicated in blue. Putative LOF mutations are highlighted in red. (PDF 211 kb) [file 12885_2019_5369_MOESM4_ESM.pdf]

Xenopus_tropicalis	-----MKMVSSDVRDKTVRKHVEHCCFL-VIVVIRMVPGFAST---VDP-TLMPIVNEEY	50	amphibian
Thamnophis_sirtalis	-----------------MNKKSSG-YFLLIILLIRMAPSFPTTVSYVDP-TLESM--EYS	39	reptiles
Anolis_carolinensis	-----------------MDKKNNGCCLL-MILLIRMAPTLSSTVSYTDP-TLAPVATEHS	41	
Serinus_canaria	-----------------MKKTTQSCFLLI-ILLTRIVPSLSSAVNYTDT-TLEPVVTENS	41	
Corvus_brachyrhynchos	-----------------MKKTTQSCFLLI-ILLTRIVPSLSSTVNYTDP-TLEPVVTENS	41	
Ficedula_albicollis	-----------------MKKTTQTCFLLI-ILLTRIVPSLSSTVNYTDP-TLEPVVTENS	41	
Anser_cygnoides_domesticus	-----------------MKKTTHSCFLLI-ILLIRMVPSLTATVNYTDP-TLEPVVTENS	41	
Anas_platyrhynchos	-----------------MKKTTHGCFLLI-ILLIRMVPSLTATVNYTDP-TLEPVVTENS	41	
Apteryx_australis_mantelli	-----------------MKKTTHSCFLLI-ILLIRMVPSLTSTVNYTDP-TLEPVVTENS	41	
Gallus_gallus	-----------------MKKTTHSCFLLI-ILLIRMVPSLTATVNYTDP-TLEPVVTENS	41	
Picoides_pubescens	-----------------MKKTTHGCFLLV-ILLIRMVPSLSSTVNYTDT-TLEPLVTENS	41	
Chaetura_pelagica	-----------------MKKTTQSCILLI-ILLIRMVPSLSSTVNYTDP-TLEPVVTENS	41	
Apaloderma_vittatum	-----------------MKKRTHSCFLLI-ILLTRMVPSLSSTVNYTDP-TLEPVVTENS	41	
Balearica_regulorum_gibbericeps	-----------------MKKTTHSCFLLI-ILLIRMIPSLSSTVNYTDP-TLEPVVTENS	41	aves
Cuculus_canorus	-----------------MKTTTHSCFLLV-ILLIKMVPTLSSTVNYTDP-TLEPVVTENS	41	
Caprimulgus_carolinensis	-----------------MKKTTHSCFLL-IILLMRMVPSLSSTVNYTDP-TLEPVVTENS	41	
Calypte_anna	-----------------MKKTAHSCILL-IILLIRMAPSLSSTVNYTDP-TLEPVVTENS	41	
Pygoscelis_adeliae	-----------------MKKTTHSCLLV-IILLIRMVPSPSSTVNYTDP-TLEPVVTENS	41	
Egretta_garzetta	-----------------MKKTTHICFLL-VILLIRMVPSLSSTVNYTDP-TLEPVVNENS	41	
Nipponia_nippon	-----------------MKKVTHS-FFLLIILLIRMVPSLSSTVNYTDP-TLEPVVTENS	41	
Charadrius_vociferus	-----------------------------------MVPSLSSTVNYTDP-TLEPVVTENS	24	
Aquila_chrysaetos_canadensis	-----------------MKKTTHSCFLLLIIFLTRMVPSLSSTVNYTDP-TLEPVVTENS	42	
Phalacrocorax_carbo	-----------------------------------MVPSLSSTVNYTDP-TLEPVATENS	24	
Sus_scrofa	-----------------MRFTLTRWCLTLFIFLNRPLPVLPDSADGAHTPTLEPEPFLYI	43	
Mus_musculus	-----------------MRFLLVNRFTLLLLLLVSPTPVLQAPTNLTDS-GLDQEPFLYL	42	
Rattus_norvegicus	MTPRRSRMKRRNLRKPKMRFLLLNRFTLLLLLLVSPTPVLQAPTNLTDS-GLDQEPFLYL	59	
Jaculus_jaculus	-----------------MKLSLTLRVAGLFILLNQPSPALPYFSNFTLPTNEPDEPFLYT	43	
Pteropus_vampyrus	-----------------MKFTLTRWCFVLFIFLNHPTPVLPTSSNNTYSPALESEPFLYV	43	
Cavia_porcellus	-----------------MRFTFTRQFLAFFILISNPASILPRSENLTFP-TFEPEPYLYS	42	
Galeopterus_variegatus	-----------------MRLRVTCRLLALFVLLNHPTPILPAFSNQTFP-TLDSEPFLYI	42	mammals
Oryctolagus_cuniculus	MAHLPPSRMKRDLQKPKMKFTLTWRCFALFLLLHQPTPVNPASSNDTHP-TVEPEPFLYV	59	
Colobus_angolensis_palliatus	-----------------MKFTFTSRCFALFLLLNHPIPILPAFSNQTYP-TIEPEPFLYV	42	
Homo_sapiens	-----------------MRFTFTSRCLALFLLLNHPTPILPAFSNQTYP-TIEPKPFLYV	42	
Pan_troglodytes	-----------------MRFTFTSRCLALFLLLNHPTPILPAFSNQTYP-TIEPEPFLYV	42	
Canis_lupus_familiaris	-----------------MKFTLTRRCLVLFIFLNHPTPVLPATSNDTYPPNMESEPFLYV	43	
Equus_caballus	-----------------MKFSLTRRCLVLFIFLNHPTPILPASSNDTYPPTIESEPFLYV	43	
Ceratotherium_simum_simum	-----------------MKFSLTRRCLVLFIFLNHPTPILPATSNDTYPPTIESEPFLYV	43	

```
Xenopus_tropicalis                  ISRQRIINSQFKCYEKMKKDSPYSKSGLYCNRTWDGWLCWDDTPAGINVTQNCPDYFPDF   110  amphibian
Thamnophis_sirtalis                 VIHQKIVDSQFKCYERMNRAPPYKKKGLYCNRTWDGWLCWDDTPAGEYADQNCPDYFQDF   99
Anolis_carolinensis                 AIPQKIVDSQFKCYERMNRTPPYKKKGLFCNRTWDGWLCWDDTPAGEFADQNCPDYFPDF   101  reptiles
Serinus_canaria                     VIRQKIIDSQFKCYERMNRAPPYKKKGLFCNRTWDGWLCWDDTPAGRLTAQNCPDYFPDF   101
Corvus_brachyrhynchos               VIRQKIIDSQFKCYERMNRAPPYKKKGLFCNRTWDGWLCWDDTPAGRLTAQNCPDYFPDF   101
Ficedula_albicollis                 VIRQKIIDSQFKCYERMNRAPPYKKKGLFCNRTWDGWLCWDDTPAGRLTAQNCPDYFPDF   101
Anser_cygnoides_domesticus          VIRQKIIDSQFKCYERMNRAPPYRKKGLFCNRTWDGWLCWDDTPAGRVTAQNCPDYFPDF   101
Anas_platyrhynchos                  VIRQKIIDSQFKCYERMNRAPPYRKKGLFCNRTWDGWLCWDDTPAGRITAQNCPDYFPDF   101
Apteryx_australis_mantelli          VIRQKIIDSQFKCYERMNRAPPYRKKGLFCNRTWDGWLCWDDTPAGRITAQNCPDYFPDF   101
Gallus_gallus                       VIRQKIIDSQFKCYERMNRAPPYRKKGLFCNRTWDGWLCWDDTPAGRITAQNCPDYFPDF   101
Picoides_pubescens                  VIRQKIIDSQFKCYERMNRAPPYKKKGLFCNRTWDGWLCWDDTPAGRTTAQNCPDYFPDF   101
Chaetura_pelagica                   VIRQKIIDSQFKCYERMNRAPPYKKKGLFCSRTWDGWLCWDDTPAGRITAQNCPDYFPDF   101
Apaloderma_vittatum                 VIRQKIIDSQFKCYERMNRAPPYKKKGLFCNRTWDGWLCWDDTPAGRITAQNCPDYFPDF   101
Balearica_regulorum_gibbericeps     VIRQKIIDSQFKCYERMNRAPPYKKKGLFCNRTWDGWLCWDDTPAGRVTAQNCPDYFPDF   101  aves
Cuculus_canorus                     IIRQKIIDSQFKCYERMNRAPPYKKKGLFCNRTWDGWLCWDDTPAGRITAQNCPDYFPDF   101
Caprimulgus_carolinensis            IIRQKIIDSQFKCYERMNRAPPYKKKGLFCNRTWDGWLCWDDTPAGRISAQNCPDYFPDF   101
Calypte_anna                        VIRQKIIDSQFKCYERMNKAPPYKKKGLFCNRTWDGWLCWDDTPAGRIAAQNCPDYFPDF   101
Pygoscelis_adeliae                  VIRQKIIDSQFKCYERMNRAPPYKKKGLFCNRTWDGWLCWDDTPAGRITAQNCPDYFPDF   101
Egretta_garzetta                    VIRQKIIDSQFKCYERMNRAPPYKKKGLFCNRTWDGWLCWDDTPAGRITAQNCPDYFPDF   101
Nipponia_nippon                     VIRQKIIDSQFKCYERMNRAPPYKKKGLFCNRTWDGWLCWDDTPAGRVTAQNCPDYFPDF   101
Charadrius_vociferus                VIRQKIIDSQFKCYERMNRAPPYKKKGLFCNRTWDGWLCWDDTPAGRITAQNCPDYFPDF   84
Aquila_chrysaetos_canadensis        VIRQKIIDSQFKCYERMNRAPPYKKKGLFCNRTWDGWLCWDDTPAGRITAQNCPDYFPDF   102
Phalacrocorax_carbo                 VIRQKIIDSQFKCYERMNRAPPYKKKGLFCNRTWDGWLCWDDTPAGRITAQNCPDYFPDF   84
Sus_scrofa                          LGKQRMLEAQHRCYDRMQKLPPYQGEGLYCNRTWDGWSCWDDTPAGVLAEQYCPDYFPDF   103
Mus_musculus                        VGRKKLLDAQYKCYDRIHQLPSYEGEGLYCNRTWDGWMCWDDTPAGATAYQHCPDYFPDF   102
Rattus_norvegicus                   VGRKKLLDAQYKCYDRIQQLPPYEGEGPYCNRTWDGWMCWDDTPAGVMSYQHCPDYFPDF   119
Jaculus_jaculus                     VGRQKLIDAQYKCYHRMEQLPPYEGEGPYCNRTWDGWMCWDDTPAGVMAYQLCPDYFPDF   103
Pteropus_vampyrus                   IGRRKMMDAENKCYRRMQQLPPFQGEGPYCNRTWDGWLCWDDTPAGVVTQQHCPDYFPDF   103
Cavia_porcellus                     VGRKKLVDAQYRCYDRMQQLPPYEGEGPYCNRTWDGWMCWDDTPAGVLSVQLCPDYFPDF   102
Galeopterus_variegatus              VGRKKLLEAQYKCYDRMQQLPPYEGEGPYCNRTWDGWMCWDDTPAGVMTYQFCPDYFPDF   102  mammals
Oryctolagus_cuniculus               IGRKKLMDAQYKCYDRMEQLPPYQGEGPYCNRTWDGWMCWDDTPAGVLSFQYCPDYFPDF   119
Colobus_angolensis_palliatus        VGRKKMMDAQYKCYDRMQQLPPYQGEGPYCNRTWDGWLCWDDTPAGVLSYQFCPDYFPDF   102
Homo_sapiens                        VGRKKMMDAQYKCYDRMQQLPAYQGEGPYCNRTWDGWLCWDDTPAGVLSYQFCPDYFPDF   102
Pan_troglodytes                     VGRKKMMDAQYKCYDRMQQLPAYQGEGPYCNRTWDGWLCWDDTPAGVLSYQFCPDYFPDF   102
Canis_lupus_familiaris              LGRKKMSDAQYKCYDRMQQLPPYQGEGPYCNRTWDGWLCWDDTPAGVLSHQYCPDYFPDF   103
Equus_caballus                      LGRKRMMDAQYKCYDRMKQLPPYQGEGPYCNRTWDGWLCWDDTPAGVLSYQYCPDYFPDF   103
Ceratotherium_simum_simum           LGRKRMMDAQYKCYDRMKQLPPYQGEGPYCNRTWDGWLCWDDTPAGVLSHQYCPDYFPDF   103
                                       ::: ::: :** ::.:   :  .* :*.****** ********    * ***** **
```

```
Xenopus_tropicalis                      DPTERASKYCDENGNWFQHPESNRTWSNYTLCNSFTSEKLKMAYILYYMAIVGHALSIVS   170   amphibian
Thamnophis_sirtalis                     DPTEIATKYCDKTGTWFRHPETNRTWSNYKRCNSFTNEKRKMIFIVYYMTIAGHVLSMVS   159   reptiles
Anolis_carolinensis                     DPTETATKYCDKTGTWFRHPESNRTWSNYTRCNSFTNEKRKMVFILYYMTIVGHALSITS   161
Serinus_canaria                         DPTERASKYCDETGNWFRHPESNRTWSNYTLCNSFTSEKLKMAFILYYMAIVGHALSITS   161   aves
Corvus_brachyrhynchos                   DPTERASKYCDETGNWFRHPESNRTWSNYTLCNSFTSEKLKMAFILYYMAIVGHALSITS   161
Ficedula_albicollis                     DPTERASKYCDETGNWFRHPESNRTWSNYTLCNSFTSEKLKMAFILYYMAIVGHALSITS   161
Anser_cygnoides_domesticus              DPTERASKYCDETGNWFRHPESNRTWSNYTLCNSFTSEKLKMAFILYYMAIVGHALSITS   161
Anas_platyrhynchos                      DPTERASKYCDETGNWFRHPESNRTWSNYTLCNSFTSEKLKMAFILYYMAIVGHALSITS   161
Apteryx_australis_mantelli              DPTERASKYCDETGNWFRHPESNRTWSNYTLCNSFTSEKLKMAFILYYMAIVGHALSITS   161
Gallus_gallus                           DPTERASKYCDETGNWFRHPESNRTWSNYTLCNSFTSEKLKMAFILYYMAIVGHALSITS   161
Picoides_pubescens                      DPTERASKYCDETGNWFRHPESNRTWSNYTLCNSFTSEKLKMAFILYYMAIVGHALSITS   161
Chaetura_pelagica                       DPTERASKYCDENGNWFRHPESNRTWSNYTLCNSFTSEKLKMAFILYYMAIVGHALSITS   161
Apaloderma_vittatum                     DPTERASKYCDETGNWFRHPESNRTWSNYTLCNSFTSEKLKMAFILYYMAIVGHALSITS   161
Balearica_regulorum_gibbericeps         DPTERASKYCDETGNWFRHPESNRTWSNYTLCNSFTSEKLKMAFILYYMAIVGHALSIAS   161
Cuculus_canorus                         DPTERASKYCDETGNWFRHPESNRTWSNYTLCNSFTSEKLKMAFILYYMAIVGHALSIAS   161
Caprimulgus_carolinensis                DPTERASKYCDETGNWFRHPESNRTWSNYTLCNSFTSEKLKMAFILYYMAIVGHALSITS   161
Calypte_anna                            DPTERASKYCDETGNWFRHPESNRTWSNYTLCNSFTSEKLKMAFILYYMAIVGHALSITS   161
Pygoscelis_adeliae                      DPTERASKYCDETGNWFRHPESNRTWSNYTLCNSFTSEKLKMAFILYYMAIVGHALSITS   161
Egretta_garzetta                        DPTERASKYCDETGNWFRHPESNRTWSNYTLCNSFTSEKLKMAFILYYMAIVGHALSITS   161
Nipponia_nippon                         DPTERASKYCDETGNWFRHPESNRTWSNYTLCNSFTSEKLKMAFILYYMAIVGHALSITS   161
Charadrius_vociferus                    DPTERASKYCDETGNWFRHPESNRTWSNYTLCNSFTSEKLKMAFILYYMAIVGHALSITS   144
Aquila_chrysaetos_canadensis            DPTERASKYCDETGNWFRHPESNRTWSNYTLCNSFTSEKLKMAFILYYMAIVGHALSITS   162
Phalacrocorax_carbo                     DPTERASKYCDETGNWFRHPESNRTWSNYTLCNSFTSEKLKMAFILYYMAIVGHALSITS   144
Sus_scrofa                              DAAEKVTKYCGEDGDWYRHPESNISWSNYTMCNAFTPDKLQNAYILYYLAIVGHSLSILT   163   mammals
Mus_musculus                            DTAEKVSKYCDENGEWFRHPDSNRTWSNYTLCNAFTSEKLQNAYVLYYLALVGHSLSIAA   162
Rattus_norvegicus                       DPTEKVSKYCDENGEWFRHPDSNRTWSNYTLCNAFTPDKLHNAYVLYYLALVGHSMSIAA   179
Jaculus_jaculus                         DPAESVTKYCDENGDWFKHPDSNRTWSNYTMCNAFTPDKLQNAYVLYYLALVGHSLSIFT   163
Pteropus_vampyrus                       DPTEKATRYCDENGDWYKHPEYNKTWSNYSMCNAFTPEKLKNAYVLYYLAIVGHSVSIAT   163
Cavia_porcellus                         DPTEKVTKYCDESGVWFKHPENNRTWSNYTLCNAFTPEKLQNAYVLYYLAIVGHSMSIIT   162
Galeopterus_variegatus                  DPTEKVTKYCDEKGVWFKHPENNRTWSNYTMCNAFTPEKLNNAYVLYYLAIVGHSLSIFT   162
Oryctolagus_cuniculus                   DPTEKVTKYCDETGVWFKHPGNNQTWSNYTMCNAFTPEKLQNAYVLYYLAIVGHSLSIFT   179
Colobus_angolensis_palliatus            DPSEKVTKYCDENGVWFKHPENNRTWSNYTMCNAFTPEKLKNAYVLYYLAIVGHSLSIFT   162
Homo_sapiens                            DPSEKVTKYCDEKGVWFKHPENNRTWSNYTMCNAFTPEKLKNAYVLYYLAIVGHSLSIFT   162
Pan_troglodytes                         DPSEKVTKYCDEKGVWFKHPENNRTWSNYTMCNAFTPEKLKNAYVLYYLAIVGHSLSIFT   162
Canis_lupus_familiaris                  DPSEKVTKYCDEKGDWYKHPENNRTWSNYTMCNAFTPEKLKNAYVLYYLAIVGHSLSIFT   163
Equus_caballus                          DPAEKVTKYCDEKGDWFKHPENNRTWSNYTMCNAFTPEKLKNAYVLYYLAIVGHSLSIFT   163
Ceratotherium_simum_simum               DPAEKVTKYCDEKGVWFKHPENNRTWSNYTMCNAFTPEKLKNAYVLYYLAIVGHSLSIFT   163
                                        * :* .::** : * *::**  * :****. **:** :* .  :::**:::.** :*: :

                                                                                      TMI
```

```
Xenopus_tropicalis               LMISLGIFFYFKSLSCQRITLHKNLFTSYVLNSVFTIVHLTAVVPDTDLVRSDPVSCKVL   230  amphibian
Thamnophis_sirtalis              LLISLGIFFYFKSLSCQRITLHKNLFCSYVLNSVFTLAHLIAIVPDQELVKKDPISCKVL   219  reptiles
Anolis_carolinensis              LLISLGIFFYFKSLSCQRITLHKNLFFSYVLNSVFTLAHLIAVVSDRDLVKNDPVSCKVL   221
Serinus_canaria                  LLISLAIFFYFKSLSCQRITLHKNLFFSYVLNSMFTIAHLIAVVPNPGLVKRDPVSCKVL   221
Corvus_brachyrhynchos            LLISLAIFFYFKSLSCQRITLHKNLFFSYVLNSMFTIAHLIAVVPNPGLVKRDPVSCKVL   221
Ficedula_albicollis              LLISLAIFFYFKSLSCQRITLHKNLFFSYVLNSMFTIAHLIVVVPNPGLVKRDPVSCKVL   221
Anser_cygnoides_domesticus       LLISLAIFFYFKSLSCQRITLHKNLFFSYVLNSVFTIAHLIAVVPNPGLVKRDPVSCKVL   221
Anas_platyrhynchos               LLISLAIFFYFKSLSCQRITLHKNLFFSYVLNSVFTIAHLIAVVPNPGLVKRDPVSCKVL   221
Apteryx_australis_mantelli       LLISLAIFFYFKSLSCQRITLHKNLFFSYVLNSVFTIAHLIAVVPNPGLVKRDPVSCKVL   221
Gallus_gallus                    LLISLAIFFYFKSLSCQRITLHKNLFFSYVLNSMFTIAHLIIVVPNPGLVKRDPVSCKVL   221
Picoides_pubescens               LLISLAIFFYFKSLSCQRITLHKNLFFSYVLNSVFTIAHLIAVVPNPGLVKRDPVSCKVL   221
Chaetura_pelagica                LLISLAIFFYFKSLSCQRITLHKNLFFSYVLNSVFTIAHLIAVVPNPGLVKRDPVSCKVL   221
Apaloderma_vittatum              LLISLAIFFYFKSLSCQRITLHKNLFCSYVLNSVFTIAHLIAVVPNPGLVKRDPVSCKVL   221  aves
Balearica_regulorum_gibbericeps  LLISLAIFFYFKSLSCQRITLHKNLFFSYVLNSMFTIAHLIAVVPNPGLVKRDPVSCKVL   221
Cuculus_canorus                  LLISLAIFFYFKSLSCQRITLHKNLFFSYVLNSMFTIAHLIAVVPNPGLVKRDPVSCKVL   221
Caprimulgus_carolinensis         LLISLAIFFYFKSLSCQRITLHKNLFFSYVLNSMFTIAHLIAVVPNPGLVKRDPVSCKVL   221
Calypte_anna                     LLISLAIFFYFKSLSCQRITLHKNLFFSYVLNSMFTIAHLIAVVPNPGLVKRDPVSCKVL   221
Pygoscelis_adeliae               LLISLAIFFYFKSLSCQRITLHKNLFFSYVLNSVFTIAHLIAVVPNPGLVKRDPVSCKVL   221
Egretta_garzetta                 LLISLAIFFYFKSLSCQRITLHKNLFFSYVLNSVFTIAHLIAVVPNPGLVKRDPVSCKVL   221
Nipponia_nippon                  LLISLAIFFYFKSLSCQRITLHKNLFFSYVLNSVFTIAHLIAVVPNPGLVKRDPVSCKVL   221
Charadrius_vociferus             LLISLAIFFYFKSLSCQRITLHKNLFFSYVLNSVFTIAHLIAVVPNPGLVKRDPVSCKVL   204
Aquila_chrysaetos_canadensis     LLISLAIFFYFKSLSCQRITLHKNLFFSYVLNSMFTIAHLIAVVPNPGLVKRDPVSCKVL   222
Phalacrocorax_carbo              LLISLAIFFYFKSLSCQRITLHKNLFFSYVLNSMFTIAHLIAVVPNPDLVKRDPVSCKVL   204
Sus_scrofa                       LLISLGIFMFLRSISCQRVTLHKNMFLTYVLNSIIIIVHLVVIVPNGELVKRDPPICKVL   223
Mus_musculus                     LVASMLIFWIFKNLSCQRVTLHKHMFLTYILNSIIIIIHLVEVVPNGDLVRRDPISCKVL   222
Rattus_norvegicus                LIASMGIFLFFKNLSCQRVTLHKNMFLTYILNSIIIIIHLVEVVPNGDLVRRDPISCKIL   239
Jaculus_jaculus                  LVISLGIFVCFRSLSCQRVTLHKHMFLTYILNSMIIIIHLVEVIPNGDLVRRDPVSCKIL   223
Pteropus_vampyrus                LVISLGIFMYFKSLGCQRVTLHKHMFLTYILNSMIIIIHLVEVVPNGELVRQDPVSCKIL   223
Cavia_porcellus                  LVVSLGIFVYFRSLGCQRVTLHKNMFLTYILNSMIIIIHLVEVVPNGELVRKDPVSCKIL   222
Galeopterus_variegatus           LVISLGIFMFYRNLSCQRVTLHKNMFLTYILNSMIIIIHLVEVVPNGDLVRKDPVSCKIL   222  mammals
Oryctolagus_cuniculus            LVISLGIFMCFRSLGCQRVTLHKNMFLTYILNSMIIIIHLVEVVPNGELVRRDPVSCKVL   239
Colobus_angolensis_palliatus     LVISLGIFVFFKSLGCQRVTLHKNMFLTYILNSMIIIIHLVEVVPNGELVRRDPVSCKIL   222
Homo_sapiens                     LVISLGIFVFFRSLGCQRVTLHKNMFLTYILNSMIIIIHLVEVVPNGELVRRDPVSCKIL   222
Pan_troglodytes                  LVISLGIFVFFRSLGCQRVTLHKNMFLTYILNSMIIIIHLVEVVPNGELVRRDPVSCKIL   222
Canis_lupus_familiaris           LVISLGIFVFFKSLGCQRVTLHKNMFLTYILNSMIIIIHLVEVVPNGELVRRDPLSCKIL   223
Equus_caballus                   LVISLGIFMFFKSLGCQRVTLHKNMFLTYILNSMIIIIHLVEVVPNGELVRRDPVSCKIL   223
Ceratotherium_simum_simum        LVISLGIFVFFKSLGCQRVTLHKNMFLTYILNSMIIIIHLVEVVPNGELVRRDPVSCKIL   223
                                 *: *: **   :.:.***:****.:* :*:***:: : **  :: :  **: **  **:*

                                    TMI                     TMII
```

Xenopus_tropicalis	QFFSQYMLGCNYFWMLCEGIYLHTLIVVAVFAEEQHLHWYYLLGWGFPLVPASIHAFART	290	amphibian
Thamnophis_sirtalis	QFFHQYTMGCNYFWMLCEGIYLHTLIVVAVFAEEQRLHWYYFLGWGFPLVPASIHAVARA	279	reptiles
Anolis_carolinensis	QFFHQYMMGCNYFWMLCEGIYLHTLIVVAVFAEEQRLHWYYLLGWGFPLVPASIHAVART	281	
Serinus_canaria	QFFHQYMLGCNYFWMLCEGIYLHTLIVVAVFAEEQRLHWYYLLGWGFPLVPASIHAVARA	281	aves
Corvus_brachyrhynchos	QFFHQYMLGCNYFWMLCEGIYLHTLIVVAVFAEEQRLHWYYLLGWGFPLVPASIHAVARA	281	
Ficedula_albicollis	QFFHQYMLGCNYFWMLCEGIYLHTLIVVAVFAEEQRLHWYYLLGWGFPLVPASIHAVARA	281	
Anser_cygnoides_domesticus	QFFHQYMLGCNYFWMLCEGIYLHTLIVVAVFAEEQRLHWYYLLGWGFPLVPASIHAVARA	281	
Anas_platyrhynchos	QFFHQYMLGCNYFWMLCEGIYLHTLIVVAVFAEEQRLHWYYLLGWGFPLVPASIHAVARA	281	
Apteryx_australis_mantelli	QFFHQYMLGCNYFWMLCEGIYLHTLIVVAVFAEEQRLHWYYLLGWGFPLVPASIHAIARA	281	
Gallus_gallus	QFFHQYMLGCNYFWMLCEGIYLHTLIVVAVFAEEQRLHWYYLLGWGFPLVPASIHAVARA	281	
Picoides_pubescens	QFFHQYMLGCNYFWMLCEGIYLHTLIVVAVFAEEQRLHWYYLLGWGFPLVPASIHAVARA	281	
Chaetura_pelagica	QFFHQYMLGCNYFWMLCEGIYLHTLIVVAVFAEEQRLHWYYLLGWGFPLVPASIHAIARA	281	
Apaloderma_vittatum	QFFHQYMLGCNYFWMLCEGIYLHTLIVVAVFAEEQRLHWYYLLGWGFPLVPASIHAVARA	281	
Balearica_regulorum_gibbericeps	QFFHQYMLGCNYFWMLCEGIYLHTLIVVAVFAEEQRLHWYYLLGWGFPLVPASIHAVARA	281	
Cuculus_canorus	QFFHQYMLGCNYFWMLCEGIYLHTLIVVAVFAEEQRLHWYYLLGWGFPVVPASIHAVARA	281	
Caprimulgus_carolinensis	QFFHQYMLGCNYFWMLCEGIYLHTLIVVAVFAEEQRLHWYYLLGWGFPLVPASIHAVARA	281	
Calypte_anna	QFFHQYMLGCNYFWMLCEGIYLHTLIVVAVFAEEQRLHWYYLLGWGFPLVPASIHAVARA	281	
Pygoscelis_adeliae	QFFHQYMLGCNYFWMLCEGIYLHTLIVVAVFAEEQRLHWYYLLGWGFPLVPASIHAVARA	281	
Egretta_garzetta	QFFHQYMLGCNYFWMLCEGIYLHTLIVVAVFAEEQRLHWYYLLGWGFPLVPASIHAVARA	281	
Nipponia_nippon	QFFHQYMLGCNYFWMLCEGIYLHTLIVVAVFAEEQRLHWYYLLGWGFPLVPASIHAVARA	281	
Charadrius_vociferus	QFFHQYMLGCNYFWMLCEGIYLHTLIVVAVFAEEQRLHWYYLLGWGFPLVPASIHAVARA	264	
Aquila_chrysaetos_canadensis	QFFHQYMLGCNYFWMLCEGIYLHTLIVVAVFAEEQRLHWYYLLGWGFPLVPASIHAVARA	282	
Phalacrocorax_carbo	QFFHQYMLGCNYFWMLCEGIYLHTLIVVAVFAEEQRLHWYYLLGWGFPLVPASIHAVARA	264	
Sus_scrofa	HFFHQYMMSCNYFWMLCEGVYLHTLIVVSVFAEGQRLWWYHVLGWGFPLIPTTAHAITRA	283	mammals
Mus_musculus	HFLHQYMMSCNYFWMLCEGIYLHTLIVMAVFTDEQRLRWYYLLGWGFPIVPTIIHAITRA	282	
Rattus_norvegicus	HFFHQYMMACNYFWMLCEGIYLHTLIVMAVFTEDQRLRWYYLLGWGFPIVPTIIHAITRA	299	
Jaculus_jaculus	HFFHQYMMACNYFWMLCEGIYLHTLIVVSVFTEEQRLRYYYFLGWGFPLVPTVIHAITRA	283	
Pteropus_vampyrus	HFFHQYMMACNYFWMLCEGIYLHTLIVVAVFAEKQHMRWYYLLGWGFPLVPTTIHAVTRA	283	
Cavia_porcellus	HFFHQYMMACNYFWMLCEGIYLHTLIVVSVFNEAKHLRWYYLLGWGFPLVPTTIHAITRA	282	
Galeopterus_variegatus	HFFHQYMMACNYFWMLCEGIYLHTLIVVAVFSGEQHLRWYYLLGWGFPLVPTTIHAITRA	282	
Oryctolagus_cuniculus	HFFHQYMMSCNYFWMLCEGIYLHTLIVVAVFAKQQHLRWYYLLGWGFPLVPTTIHAITRA	299	
Colobus_angolensis_palliatus	HFFHQYMMACNYFWMLCEGIYLHTLIVMAVFTEKQRLRWYYLLGWGFPLVPTTIHAITRA	282	
Homo_sapiens	HFFHQYMMACNYFWMLCEGIYLHTLIVVAVFTEKQRLRWYYLLGWGFPLVPTTIHAITRA	282	
Pan_troglodytes	HFFHQYMMACNYFWMLCEGIYLHTLIVVAVFTEKQRLRWYYLLGWGFPLVPTTIHAITRA	282	
Canis_lupus_familiaris	HFFHQYMMACNYFWMLCEGIYLHTLIVVAVFTEEQHLRWYYLLGWGFPLVPTTIHAITRA	283	
Equus_caballus	HFFHQYMMACNYFWMLCEGIYLHTLIVVAVFTEEQRLRWYYLLGWGFPLVPTTIHAITRA	283	
Ceratotherium_simum_simum	HFFHQYMMACNYFWMLCEGIYLHTLIVVAVFTEEQRLRWYYLLGWGFPLVPTTIHAITRA	283	
	:*: ** :.**********:*******::** ::: :*:.******::*: **.:*:		

TMIII TMIV

Xenopus_tropicalis	KYFNDNCWMSVETHLLYIVHGPIMAALLVNLFFLLNIVLVLVTKLRDTHRAESNMYMKAV	350	amphibians
Thamnophis_sirtalis	RYFNDNCWISVDTYLLYIVHGPVMAALLVNFFFLLNIVRVLVTKLRDTHRAESNMYMKAV	339	
Anolis_carolinensis	KYFNDNCWISVDTHLLYVVHGPVMAALLVNFFFLLNIVRVLVTKLRDTHRAESNMYMKAV	341	reptiles
Serinus_canaria	RYFNDNCWMSVDTHLLYIVHGPVMAALLVNFFFLLNIVRVLVTKLRDTHRAESNMYMKAV	341	
Corvus_brachyrhynchos	RYFNDNCWMSVDTYLLYIVHGPVMAALLVNFFFLLNIVRVLVTKLRDTHRAESNMYMKAV	341	aves
Ficedula_albicollis	RYFNDNCWMSVDTHLLYIVHGPVMAALLVNFFFLLNIVRVLVTKLRDTHRAESNMYMKAV	341	
Anser_cygnoides_domesticus	KYFNDNCWMSVDTYLLYIVHGPVMAALLVNFFFLLNIVRVLVTKLRDTHRAESNMYMKAV	341	
Anas_platyrhynchos	KYFNDNCWMSVDTYLLYIVHGPVMAALLVNFFFLLNIVRVLVTKLRDTHRAESNMYMKAV	341	
Apteryx_australis_mantelli	KYFNDNCWMSVDTHLLYIVHGPVMAALLVNFFFLLNIVRVLVTKLRDTHRAESNMYMKAV	341	
Gallus_gallus	KYFNDNCWMSVDTHLLYIVHGPVMAALLVNFFFLLNIVRVLVTKLRDTHRAESNMYMKAV	341	
Picoides_pubescens	KYFNDNCWMSVDTHLLYIVHGPVMAALLVNFFFLLNIVRVLVTKLRDTHRAESNMYMKAV	341	
Chaetura_pelagica	KYFNDNCWMSVDTHLLYIVHGPVMAALLVNFFFLLNIVRVLVTKLRDTHRAESNMYMKAV	341	
Apaloderma_vittatum	RYFNDNCWMSVDTHLLYIVHGPVMAALLVNFFFLLNIVRVLVTKLRDTHRAESNMYMKAV	341	
Balearica_regulorum_gibbericeps	KYFNDNCWMSVDTHLLYIVHGPVMAALLVNFFFLLNIVRVLVTKLRDTHRAESNMYMKAV	341	
Cuculus_canorus	KYFNDNCWMSVDTHLLYIVHGPVMAALLVNFFFLLNIVRVLVTKLRDTHRAESNMYMKAV	341	
Caprimulgus_carolinensis	KYFNDNCWMSVDTHLLYIVHGPVMAALLVNFFFLLNIVRVLVTKLRDTHRAESNMYMKAV	341	
Calypte_anna	KYFNDNCWMSVDTHLLYIVHGPVMAALLVNFFFLLNIVRVLVTKLRDTHRAESNMYMKAV	341	
Pygoscelis_adeliae	RYFNDNCWMSVDTHLLYIVHGPVMAALLVNFFFLLNIVRVLVTKLRDTHRAESNMYMKAV	341	
Egretta_garzetta	RYFNDNCWMSVDTHLLYIVHGPVMAALLVNFFFLLNIVRVLVTKLRDTHRAESNMYMKAV	341	
Nipponia_nippon	RYFNDNCWMSVDTHLLYIVHGPVMAALLVNFFFLLNIVRVLVTKLRDTHRAESNMYMKAV	341	
Charadrius_vociferus	KYFNDNCWMSVDTHLLYIVHGPVMAALLVNFFFLLNIVRVLVTKLRDTHRAESNMYMKAV	324	
Aquila_chrysaetos_canadensis	RYFNDNCWMSVDTHLLYIVHGPVMAALLVNFFFLLNIVRVLVTKLRDTHRAESNMYMKAV	342	
Phalacrocorax_carbo	RYFNDNCWMSVDTHLLYIVHGPVMAALLVNFFFLLNIVRVLVTKLRDTHRAESNMYMKAV	324	
Sus_scrofa	VLFNDNCWLSVDTNLLYIIHGPVMAALVVNFFFLLNILRVLVKKLKESQEAESHMYLKAV	343	mammals
Mus_musculus	LYYNDNCWLSAETHLLYIIHGPVMVALVVNFFFLLNIVRVLVTKMRQTHEAESYMYLKAV	342	
Rattus_norvegicus	VYYNDNCWLSTETHLLYIIHGPVMAALVVNFFFLLNIVRVLVTKMRQTHEAEAYMYLKAV	359	
Jaculus_jaculus	LYYDDNCWLSVETHLLYIIHGPVMAALVVNFFFLLNIVRVLVTKMRETHEAESHMYLKAV	343	
Pteropus_vampyrus	LYFNDNCWLSVETYLLYIIHGPVMAALVVNFFFLLNIVRVLVKKMRETQEVDSHMYLKAV	343	
Cavia_porcellus	LYFNDNCWISVDTHLLYIIHGPVMVALVVNFFFLLNIVRVLVTKMRETHEAESYMYLKAV	342	
Galeopterus_variegatus	LYFNDNCWLSVETHLLYIIHGPVMAALVVNFFFLLNIVRVLVTKMRETHEAESQMYLKAV	342	
Oryctolagus_cuniculus	IYFNDNCWMSVETHLLYIIHGPVMAALVVNFFFLLNIVRVLVTKMRETHEAESHMYLKAV	359	
Colobus_angolensis_palliatus	VYFNDNCWLSVETHLLYIIHGPVMAALVVNFFFLLNIVRVLVTKMRETHEAESHMYLKAV	342	
Homo_sapiens	VYFNDNCWLSVETHLLYIIHGPVMAALVVNFFFLLNIVRVLVTKMRETHEAESHMYLKAV	342	
Pan_troglodytes	VYFNDNCWLSVETHLLYIIHGPVMAALVVNFFFLLNIVRVLVTKMRETHEAESHMYLKAV	342	
Canis_lupus_familiaris	LYFNDNCWLSVETHLLYIIHGPVMAALVVNFFFLLNIVRVLVSKMRETQEAESHMYLKAV	343	
Equus_caballus	LYFNDNCWLSVETHLLYIIHGPVMAALVVNFFFLLNIVRVLVSKMRETQEAESHMYLKAV	343	
Ceratotherium_simum_simum	LYFNDNCWLSVETHLLYIIHGPVMAALVVNFFFLLNIVRVLVSKMRETQEAESHMYLKAV	343	
	::****:*.:* ***::***:*.**:**:******: ***.*:::::..:: **:***		

TMV TMVI

```
Xenopus_tropicalis                    RATLILVPLLGIQFVIFPWRPDTRLAGEIYDYIMNILMHYQGLLVATIFCFFNGEVQGAL   410  ] amphibians
Thamnophis_sirtalis                   RATLILVPLLGIQFVIFPWRPENKLAGEIYDYIMHILMHYQGLLVATIFCFFNGEVQGTL   399  ]
Anolis_carolinensis                   RATLILVPLLGIQFVIIPWRPENKLAGEVYDYIMHILMHYQGLLVATIFCFFNGEVQGAL   401  ] reptiles
Serinus_canaria                       RATLILVPLLGIQFVIIPWRPENRLAGEIYDYIMHILMHYQGLLVATIFCFFNGEVQGAL   401  ]
Corvus_brachyrhynchos                 RATLILVPLLGIQFVIIPWRPENRLAGEIYDYIMHILMHYQGLLVATIFCFFNGEVQGAL   401  ]
Ficedula_albicollis                   RATLILVPLLGIQFVIIPWRPENRLAGEIYDYIMHILMHYQGLLVATIFCFFNGEVQGAL   401  |
Anser_cygnoides_domesticus            RATLILVPLLGIQFVIIPWRPENRLAGEIYDYIMHILMHYQGLLVATIFCFFNGEVQGAL   401  |
Anas_platyrhynchos                    RATLILVPLLGIQFVIIPWRPENRLAGEIYDYIMHILMHYQGLLVATIFCFFNGEVQGAL   401  |
Apteryx_australis_mantelli            RATLILVPLLGIQFVIIPWRPENRLAGEIYDYIMHILMHYQGLLVATIFCFFNGEVQGAL   401  |
Gallus_gallus                         RATLILVPLLGIQFVIIPWRPENRLAGEIYDYIMHILMHYQGLLVATIFCFFNGEVQGAL   401  |
Picoides_pubescens                    RATLILVPLLGIQFVIIPWRPENRLAGEIYDYIMHILMHYQGLLVATIFCFFNGEVQGAL   401  |
Chaetura_pelagica                     RATLILVPLLGIQFVIIPWRPENRLAGEIYDYIMHILMHYQGLLVATIFCFFNGEVQGAL   401  |
Apaloderma_vittatum                   RATLILVPLLGIQFVIIPWRPENRLAGEIYDYIMHILMHYQGLLVATIFCFFNGEVQGTL   401  | aves
Balearica_regulorum_gibbericeps       RATLILVPLLGIQFVIIPWRPENRLAGEIYDYIMHILMHYQGLLVATIFCFFNGEVQGAL   401  |
Cuculus_canorus                       RATLILVPLLGIQFVIIPWRPENRLAGEIYDYIMHILMHYQGLLVATIFCFFNGEVQGAL   401  |
Caprimulgus_carolinensis              RATLILVPLLGIQFVIIPWRPENRLAGEIYDYIMHILMHYQGLLVATIFCFFNGEVQGAL   401  |
Calypte_anna                          RATLILVPLLGIQFVIIPWRPENRLAGEIYDYIMHILMHYQGLLVATIFCFFNGEVQGAL   401  |
Pygoscelis_adeliae                    RATLILVPLLGIQFVIIPWRPENRLAGEIYDYIMHILMHYQGLLVATIFCFFNGEVQGAL   401  |
Egretta_garzetta                      RATLILVPLLGIQFVIIPWRPENRLAGEIYDYIMHILMHYQGLLVATIFCFFNGEVQGAL   401  |
Nipponia_nippon                       RATLILVPLLGIQFVIIPWRPENRLAGEIYDYIMHILMHYQGLLVATIFCFFNGEVQGAL   401  |
Charadrius_vociferus                  RATLILVPLLGIQFVIIPWRPENRLAGEIYDYIMHILMHYQGLLVATIFCFFNGEVQGAL   384  |
Aquila_chrysaetos_canadensis          RATLILVPLLGIQFVIIPWRPENRLAGEIYDYIMHILMHYQGLLVATIFCFFNGEVQGAL   402  |
Phalacrocorax_carbo                   RATLILVPLLGIQFVIIPWRPENRLAGEIYDYIMHILMHYQGLLVATIFCFFNGEVQGAL   384  ]
Sus_scrofa                            RATLILVPLLGVQFVVLPWRPSTPLLGKIYDYVVHSLIHFQGFFVAIIYCFCNHEVQGAL   403  ]
Mus_musculus                          KATMVLVPLLGIQFVVFPWRPSNKVLGKIYDYLMHSLIHFQGFFVATIYCFCNHEVQVTL   402  |
Rattus_norvegicus                     KATMVLVPLLGIQFVVFPWRPSNKVLGKIYDYLMHSLIHFQGFFVATIYCFCNHEVQVTL   419  |
Jaculus_jaculus                       KATMVLVPLLGIQFVVFPWRPSNKILGKIYDYLMHSLIHFQGFFVATIYCFCNNEVQTTL   403  |
Pteropus_vampyrus                     RATLILVPLLGIQFVVFPWRPSNKVLGKIYDYLMHSLIHFQGFFVAVIYCFYNSEVQTAV   403  |
Cavia_porcellus                       KATMILVPLLGIQFVVFPWRPSNKVLGKIYDYFMHSLIHFQGFFVATIYCFCNNEVQTTL   402  |
Galeopterus_variegatus                KATMILVPLLGIQFVVFPWRPSNKILGKIYDYFMHSLIHFQGFFVATIYCFCNKEVQTTV   402  | mammals
Oryctolagus_cuniculus                 KATMILVPLLGIQFVVFPWRPSNKILGKIYDYLMHSLIHFQGFFVATIYCFCNNEVQTTV   419  |
Colobus_angolensis_palliatus          KATMILVPLLGIQFVVFPWRPSNKMLGKIYDYVMHSLIHFQGFFVATIYCFCNNEVQTTV   402  |
Homo_sapiens                          KATMILVPLLGIQFVVFPWRPSNKMLGKIYDYVMHSLIHFQGFFVATIYCFCNNEVQTTV   402  |
Pan_troglodytes                       KATMILVPLLGIQFVVFPWRPSNKMLGKIYDYVMHSLIHFQGFFVATIYCFCNNEVQTTV   402  |
Canis_lupus_familiaris                RATLILVPLLGIQFVVFPWRPSNKMLGKIYDYLMHSLIHFQGFFVAVIYCFCNNEVQTTV   403  |
Equus_caballus                        RATLILVPLLGIQFVVFPWRPSNKMLGKIYDYLMHSLIHFQGFFVAMIYCFCNNEVQTTV   403  |
Ceratotherium_simum_simum             RATLILVPLLGIQFVVFPWRPSNKMLGKIYDYLMHSLIHFQGFFVAMIYCFCNNEVQTTL   403  ]
                                      :**::******:***::****.. : *::***.:. *:*:**::** *:** * *** ::

                                             TMVI                      TMVII
```

```
Xenopus_tropicalis                 KRQWMQYKTQWGQRRREHCSMRSTSYT---------ATSITEVPIYLYHHDSNSEQ--LN    459   amphibian
Thamnophis_sirtalis                KRQWMQYKTQWGQRRRDHCSTRSTSYT---------ATSITEVPVYLYHRDSNSEH--LN    448
Anolis_carolinensis                KRQWMQYKTQWGQRRREHCSTRSTSYT---------ATSITEVPIYLYHHDSSSEQ--FN    450
Serinus_canaria                    KRQWAQYKTQWGQRRREHCSTRSTSYT---------ATSITEVPVYLYHHDANNEH--LN    450
Corvus_brachyrhynchos              KRQWAQYKTQWGQRRREHCSTRSTSYT---------ATSITEVPVYLYPHDANNEQ--LN    450
Ficedula_albicollis                KRQWTQYKTQWGQRRREHCSTRSTSYT---------ATSITEVPVYLYHHDANNEQ--LN    450
Anser_cygnoides_domesticus         KRQWTQYKTQWGQRRREHCSTRSTSYT---------ATSITEVPVYLYHHDSNNEQ--LN    450
Anas_platyrhynchos                 KRQWTQYKTQWGQRRREHCSTRSTSYT---------ATSITEVPVYLYHHDSNNEQ--LN    450
Apteryx_australis_mantelli         KRQWTQYKTQWGQRRREHCSTRSTSYT---------ATSITEVPVYLYHHDSNNEQ--LN    450
Gallus_gallus                      KRQWTQYKTQWGQRRREHCSTRSTSYT---------ATSITEVPVYLYHHDSNNEQ--LN    450
Picoides_pubescens                 KRQWTQYKTQWGQRRREHCSTRSTSCT---------ATSISEVPVYLYHHDSNNEQ--FN    450
Chaetura_pelagica                  KRQWTQYKTQWGQRRREHCSTRSTSYT---------ATSITEVPVYLYHRDSSNEQ--IN    450
Apaloderma_vittatum                KRQWTQYKAQWGQRRREHCSTRSTSYT---------ATSITEVPIYLYHHESNSEQ--LN    450
Balearica_regulorum_gibbericeps    KRQWTQYKTQWGQRRREHCSTRSTSYT---------ATSITEVPVYLYHHDSNNEQ--LN    450
Cuculus_canorus                    KRQWTQYKTQWGQRRREHCSTRSTSYT---------ATSITEVPVYLYHHDSNNEQ--LN    450
Caprimulgus_carolinensis           KRQWTQYKTQWGQRRREHCSTRSTSYT---------ATSITEVPVYLFHHDSNNEQ--LN    450
Calypte_anna                       KRQWTQYKTQWGQRRREHCSTRSTSYT---------ATSITEVPVYLYHHDSNNEQ--LN    450
Pygoscelis_adeliae                 KRQWAQYKTQWGQRRREHCSTRSTSYT---------ATSITEVPVYLYHHDSTNEQ--LN    450
Egretta_garzetta                   KRQWTQYKTQWGQRRREHCSTRSTSYT---------ATSITELPVYLYHHDSNNEQ--LN    450
Nipponia_nippon                    KRQWTQYKTQWGQRRREHCSTRSTSYT---------ATSITEVPVYLYHHDSNNEQ--LN    450
Charadrius_vociferus               KRQWTQYKTQWGQRRREHCSTRSTSYT---------ATSITEVPVYLYHHDSNNEQ--LN    433
Aquila_chrysaetos_canadensis       KRQWTQYKTQWGQRRREHCSTRSTSYT---------ATSITEVPVYLYHHDSNNEQ--LN    451
Phalacrocorax_carbo                KRQWTQYKTQWGQRRREHCSTRSTSYT---------ATSITEVPVYLYHHDSNNEQ--LN    433
Sus_scrofa                         KRQWNQYQAQ---RWAGRRSTRAANAAAATAAAAAALAETVEIPVYICHQEPREEPAGEE    460
Mus_musculus                       KRQWTQFKIQWSQRWGRR-RPTN---RVVSAPRAVAFAEPDGLPIYICHQEPR-NPPISN    457
Rattus_norvegicus                  KRQWAQFKIQWSHRWGRRRRPTN---RVVSAPRAVAFAEPGGLPIYICHQEPR-NPPVSN    475
Jaculus_jaculus                    KRQWTQFKLQ---HWGTALTNRTAP-QASPAATASAEAGDIDLPVYICHREPPRA----N    455
Pteropus_vampyrus                  KRQWIQFKIQWDQRRGGRNHRRPHF-HAAAAAAAAAEAEAGDIPVYICHQEPR-NNEPAI    461
Cavia_porcellus                    KRQWAQFKIQWNQRWGTRPSNRSAA-A--RAAAAAAEAGGDNIPVYICHQE--PRNDPPN    457
Galeopterus_variegatus             KRHWAQLRIQWDQRWGPRPSARSTA-R--AA-A---SAEAGDIPVYICHPEPEPRNEPAG    455
Oryctolagus_cuniculus              KRQWVQFKIQWNQRWGRRPAHRSVS-R--TA-A---SAEEGGIPVYIYHQE--PRNDQAH    470
Colobus_angolensis_palliatus       KRQWVQFKIQWNQRWGRRPSNRSA--R--AA-A---AAEAGDIPVYICHQE--PRNEPAN    452
Homo_sapiens                       KRQWAQFKIQWNQRWGRRPSNRSA--R--AAAA---AAEAGDIPIYICHQE--LRNEPAN    453
Pan_troglodytes                    KRQWAQFKIQWNQRWGRRPSNRSA--R--AAAA---AAEAGDIPIYICHQE--PRNEPAN    453
Canis_lupus_familiaris             KRQWAQFKTQWDQRWGRPNPRRSAA-S--AAAA---AAEAGDIPVYICHQE--PRNEAAN    455
Equus_caballus                     KRHWAQFKTQWNQRWGRRTNNRSIS----NAAA---SAEAGDIPVYICHQE--PRNELAN    454
Ceratotherium_simum_simum          KRHWAQFKTQWNQRWGRRSNNRSIS----NAAA---AAGARDIPVYICHQE--PRNEPAN    454
                                   **:* * : *   :                       :    :*:*:   :
```

reptiles (Thamnophis_sirtalis, Anolis_carolinensis)

aves (Serinus_canaria – Phalacrocorax_carbo)

mammals (Sus_scrofa – Ceratotherium_simum_simum)

```
Xenopus_tropicalis                    -GKYGDESEITALNSG--DTYA 478   amphibian
Thamnophis_sirtalis                   -GKYIHDSELVALKSG--ETSA 467 ┐
Anolis_carolinensis                   -GKYIDDSELVALKSG--ETSA 469 ┘ reptiles
Serinus_canaria                       -GRYVEDSELVALKSG--DTSA 469 ┐
Corvus_brachyrhynchos                 -GRYVEDSELVALKSG--ETSA 469 │
Ficedula_albicollis                   -GRYLEDSELVALKSG--ETSA 469 │
Anser_cygnoides_domesticus            -GRYIDDSELVALKSG--ETSA 469 │
Anas_platyrhynchos                    -GRYIDDSELVALKSG--ETSA 469 │
Apteryx_australis_mantelli            -GRYIDDSELVALKSG--ETSA 469 │
Gallus_gallus                         -GRYVDDSELVALKSG--ETSA 469 │
Picoides_pubescens                    -GRYTEDSELVALKSG--ETSA 469 │
Chaetura_pelagica                     -GRYIEDSELVALKSG--ETSA 469 │
Apaloderma_vittatum                   -GRYVEDSELVALKSG--ETSA 469 │
Balearica_regulorum_gibbericeps       -GRYIEDSELVALKSG--ETSA 469 │ aves
Cuculus_canorus                       -GRYVEDSELVALKSG--ETSA 469 │
Caprimulgus_carolinensis              -GRYVEDSELVALKPG--ETSA 469 │
Calypte_anna                          -GRYTEDSELVALKSG--ETSA 469 │
Pygoscelis_adeliae                    -GRYIEDSELVALKSG--ETSA 469 │
Egretta_garzetta                      -GRYIEDSELVALKSG--ETSA 469 │
Nipponia_nippon                       -GRYAEDSELVALKSG--ETSA 469 │
Charadrius_vociferus                  -GRYVEDSELVALKSG--ETSA 452 │
Aquila_chrysaetos_canadensis          -GRYVEDSELVALKSG--ETSA 470 │
Phalacrocorax_carbo                   -GRYVEDSELVALKSG--ETSA 452 ┘
Sus_scrofa                            PVVEVEGVEVIAMEVLEQETSA 482 ┐
Mus_musculus                          -NEGEESTEMIPMNVIQQDASA 478 │
Rattus_norvegicus                     -NEGEEGTEMIPMNVIQQDSSA 496 │
Jaculus_jaculus                       -NQVLEGAEIIPLNTVEQESSA 476 │
Pteropus_vampyrus                     -NLGEEGAEDIPMEIIEQESCA 482 │
Cavia_porcellus                       -NQGEEGAEMIVLNIIEKESSA 478 │
Galeopterus_variegatus                -NEGERGAELIPLNIIEHESSA 476 │ mammals
Oryctolagus_cuniculus                 -SLGEEGAEIIPLNIIEQESSA 491 │
Colobus_angolensis_palliatus          -NQGEESAEIIPLNIIEQETSA 473 │
Homo_sapiens                          -NQGEESAEIIPLNIIEQESSA 474 │
Pan_troglodytes                       -NQGEESAEIIPLNIIEQESSA 474 │
Canis_lupus_familiaris                -NLGEEGAEVIALEIIEQESSA 476 │
Equus_caballus                        -NLGGEGAEVIALEIIEQESSA 475 │
Ceratotherium_simum_simum             -NLGGEGAEVIALEIIEQESSA 475 ┘
                                              *   ::    :: *
```
